# Metagenomic Insights into Metabolic Capacities of the Gut Microbiota in a Fungus-Cultivating Termite (*Odontotermes yunnanensis*)

**DOI:** 10.1371/journal.pone.0069184

**Published:** 2013-07-17

**Authors:** Ning Liu, Lei Zhang, Haokui Zhou, Meiling Zhang, Xing Yan, Qian Wang, Yanhua Long, Lei Xie, Shengyue Wang, Yongping Huang, Zhihua Zhou

**Affiliations:** 1 Key Laboratory of Insect Developmental and Evolutionary Biology, Institute of Plant Physiology and Ecology, Shanghai Institutes for Biological Sciences, Chinese Academy of Sciences, Shanghai, China; 2 Key Laboratory of Synthetic Biology, Institute of Plant Physiology and Ecology, Shanghai Institutes for Biological Sciences, Chinese Academy of Sciences, Shanghai, China; 3 Department of Microbiology, Li Ka Shing Institute of Health Sciences, The Chinese University of Hong Kong, Prince of Wales Hospital, Shatin, Hong Kong SAR, China; 4 Department of Biochemistry and Molecular Biology, College of Life Science, East China Normal University, Shanghai, China; 5 Department of Biochemistry, College of life science, Anhui Agricultural University, Hefei, China; 5 Chinese National Human Genome Center at Shanghai, Shanghai, China; Hospital for Sick Children, Canada

## Abstract

Macrotermitinae (fungus-cultivating termites) are major decomposers in tropical and subtropical areas of Asia and Africa. They have specifically evolved mutualistic associations with both a *Termitomyces* fungi on the nest and a gut microbiota, providing a model system for probing host-microbe interactions. Yet the symbiotic roles of gut microbes residing in its major feeding caste remain largely undefined. Here, by pyrosequencing the whole gut metagenome of adult workers of a fungus-cultivating termite (*Odontotermes yunnanensis*), we showed that it did harbor a broad set of genes or gene modules encoding carbohydrate-active enzymes (CAZymes) relevant to plant fiber degradation, particularly debranching enzymes and oligosaccharide-processing enzymes. Besides, it also contained a considerable number of genes encoding chitinases and glycoprotein oligosaccharide-processing enzymes for fungal cell wall degradation. To investigate the metabolic divergence of higher termites of different feeding guilds, a SEED subsystem-based gene-centric comparative analysis of the data with that of a previously sequenced wood-feeding *Nasutitermes* hindgut microbiome was also attempted, revealing that SEED classifications of nitrogen metabolism, and motility and chemotaxis were significantly overrepresented in the wood-feeder hindgut metagenome, while Bacteroidales conjugative transposons and subsystems related to central aromatic compounds metabolism were apparently overrepresented here. This work fills up our gaps in understanding the functional capacities of fungus-cultivating termite gut microbiota, especially their roles in the symbiotic digestion of lignocelluloses and utilization of fungal biomass, both of which greatly add to existing understandings of this peculiar symbiosis.

## Introduction

Termites are notable for extensive plant biomass degradation capacity. However, instead of providing all endogenous enzymes necessary for foodstuffs digestion, they have developed mutualistic symbiosis with versatile groups of microorganisms which are known to actually play essential roles for this process. Higher termites of the subfamily Macrotermitinae, a most prevalent and influential insect group in tropics and subtroptics of Africa and Asia [1], are especially distinguished. In addition to symbioses with gut microbes as in other guilds of termites, Macrotermitinae specifically cultivate a *Termitomyces* fungi (Class: Basidiomycetes) in their nests for both lignocellulose degradation and food. This peculiar fungiculture behavior are known to be achieved by a complex colony with elaborate caste and labor division ([2], [3], [4], [5], and references therein). Firstly, plant matters, including leaves, grasses and stalks, are collected by old workers to the nest, where young workers masticate, excreted rapidly without obvious digestion and deposited as new layer of fungus combs. Thereon, the *Termitomyces* grow and the fungus combs mature, during which young workers consume fungus nodules along with primary ingestion as a way of inoculating new parts of fungus combs, while old workers consume mature parts of fungus combs, including both fungus-processed plant matters and senescent fungal mycelia to produce final feces.

The specific involvement of *Termitomyces* fungi in Macrotermitinae has enabled them a continually prior focus of attention as to resolving the nutritional basis underlying fungus-cultivating termites. Hitherto several roles of the fungal partner have been proposed or revealed [6], [7], including lignin degradation as the fungus comb matures, provision of hydrolytic enzymes for plant cell wall degradation, and serving as high quality nourishment (fungal mycelium) for termite hosts. However a gut flora in fungus-cultivating termites dwelling in a distinctive microenvironments [8] also exists [9], [10], [11], [12], and is known to vary among ages and castes [5] and be distinct from that associated with the fungus comb [13]. Thus, a symbiotic role of the gut bacteria for fungus-growers, with special respect to the lignocellulose degradation process, is also likely, but is far from being fully studied and defined [7]. Indeed, our recent functional exploration and recovery of cellulases and hemicellulases genes from gut microbiome of a *Macrotermes* species has already shown instructive hints to this possibility [14]. However, an overall insight into the nutritional potentials of this microflora remains. Taking advantages of next-generation sequencing technologies, as was already performed for various plant biomass-degrading ecosystems such as microbiomes of a wood-feeding termite hindgut [15], bovine rumen [16], a tammar wallaby forgut [17], and leaf cutter ant gardens [18], [19], we present here the first metagenomic sequences of the gut microbiome of a fungus-cultivating termite. Based on this, degradative capacities of this gut microbiota for both dietary plant and fungal biomass for termite hosts were characterized. Furthermore, a comparative metagenomic analysis with the previously sequenced wood-feeding higher termite [15] was also performed, to shed light on the adaptation of gut microbiota to different feeding guilds of the termites hosts and to further expand our vision of the evolutionary diversified and optimized symbiotic systems in termites.

## Materials and Methods

### Ethics Statement

No specific permissions were required for sampling termites from Xishuangbanna, Yunnan Province, China. Because termites are unregulated insects widely distributed in the natural ecosystems there. And the field studies did not involve endangered or protected species.

### Termite Sampling

Old adult workers and some adult soldiers were selectively collected from a nest of *Odontotermes* in Xishuangbanna, Yunnan Province, China, in March 2008. Surface sterilization and evisceration of old adult workers were conducted following the same protocol as we previously described [14], and the collected guts were immediately stored at –80°C until DNA extraction. Meanwhile, the entire bodies of the adult soldiers were directly frozen and stored, and the heads were used for both morphological as well as mitochondrial COII gene-based molecular identification according to the methods of Ohkuma *et al.* [20]. And both morphology and sequence alignment of the COII gene of the heads of the sampled adult soldiers showed that the nest of fungus-cultivating termites studied in present work was *Odontotermes yunnanensis*.

### Termite Gut Microbial DNA Extraction

To profile the full-scale metabolic potential of the given fungus-cultivating termite gut microbiota, metagenomic DNA from nearly 2,000 whole guts (including all of the foregut, midgut, and hindgut) of the present old adult workers were extracted using an indirect method we previously described [14]. Briefly, a mild trypsin digestion step (instead of a preliminary mechanical grinding step with a 2-ml Tenbroeck tissue grinder, as used by Zhou et al. [21]) was firstly applied to disintegrate the gut tissues and release the microbial cells. Then a critical 800×g centrifugation step (instead of a preliminary 200×g step, as used during human fecal environmental DNA (eDNA) preparation [22]) was performed to pre-exclude termite gut tissues and cells. Finally, microbial cells were collected and DNA was extracted with the method of Zhou et al. [23] with minor modifications [14]. The refined fractionation procedure we applied was observed to be able to greatly reduce host eukaryotic contamination using 18S rRNA gene and its RFLP (*Afa* I and *Hin*f I) as indicator. Potential impact of the refined fractionation procedure on gut microbial community was evaluated by DGGE. And the nearly identical DGGE fingerprints of the V3 regions of 16S rRNA genes amplified from DNA extracted with and without these two refinements during extruding eukaryotic cells indicated that no obvious effect to the microbial community was detected (Figure S1 in File S1), despite a minute part of microbial cells were observed lost. The concentration and purity of DNA were analyzed by gel electrophoresis and spectrophotometric quantification.

### 454 Pyrosequencing and Sequence Analysis

A total of 5 µg of extracted DNA was subjected to one run of the 454 Life Sciences Genome Sequencer GS FLX (Roche Diagnostics). Since the length of the generated reads were short (averaging 234 base pairs), assembly of the raw sequences was tried but failed, so unassembled metagenomic dataset was subjected to further analysis. To identify potential sequences belonging to known CAZy families, the present metagenome were searched (E≤10^−4^) against the set of profile hidden Markov models (HMMs) representing the signature domain of each CAZy family defined by Yin et al. [24], as deposited on dbCAN (http://csbl.bmb.uga.edu/dbCAN/) for its latest version (dbCAN-fam-HMMs.txt.v2). Besides, to survey predicted domains often associated with GH catalytic domains, as indexed by Park et al. [25] and Warnecke et al. [15], pyrosequencing reads were also searched against the Pfam (E≤10^−4^) and the eggNOG (E≤10^−5^) [26] databases respectively. To determine the taxonomic distribution of environmental sequences in the dataset, all reads were firstly subjected to BLASTX searches against the NCBI nr database with an E-value cutoff of 10^−2^, then were phylogenetically binned according to the LCA-based algorithm implemented in MEGAN [27] with a bit-score threshold of 35.0. Given the short length of analyzed reads, the top-percentage filter was set to 90% to discard low-score matches that may be randomly aligned. Identification of 16S rRNA genes was based on a BLASTN search against the RDP database (E≤10^−5^) [28]. Hits were aligned with the NAST aligner [29], and only those with an alignment length >100 nt and a sequence identity >75% were counted. The same alignment was imported into the ARB software [30], based on which phylogenetic positions of these qualified 16S rRNA gene fragments were determined. Metagenomic reads were annotated based on BLAST searches (E≤10^−5^) against the SEED FIG families downloaded from the MGRAST server (http://metagenomics.theseed.org/) [31]. And the resulting annotated reads were defined as environmental gene tags (EGTs) for further comparative analysis.

### Statistical Analysis

To investigate the metabolic divergence of the gut microbiomes between fungus-grower and that of a previously sequenced wood-feeding *Nasutitermes* species [15], a gene-centric comparative analysis was conducted using abundance of EGTs in each of the two dataset as a comparative metric, as described and applied in other cases [16], [19], [32]. Given the largely assembled sequences from the latter, data of all its genes were downloaded from IMG (http://img.jgi.doe.gov/) under the project ID 11653, and subjected to annotation by SEED with the same parameters. The proportions of EGTs in each subsystem with respect to the total number of EGTs in that metagenome (163902 SEED annotated EGTs in the present dataset versus 36445 SEED annotated EGTs in the *Nasutitermes* dataset) were calculated and relative abundances were compared. Effect size was defined as ratio of proportions of each subsystem in individual dataset and was calculated by STAMP program as described by Parks and Beiko [33].

### Construction, Sequencing, and Phylogenetic Analysis of the 16S rRNA Gene Clone Library

16S rRNA genes were amplified with universal bacterial primers 27F (5′-AGAGTTTGATCCTGGCTCAG-3′) and 1492R (5′-TACGGYTACCTTGTTACGACTT-3′). PCR reactions were performed as previously described [13], and a ‘reconditioning PCR’ procedure was further applied to the resulting PCR products according to the protocols of Polz *et al.* [34] to minimize heteroduplexes that might be introduced by PCR reactions. 16S rRNA gene amplicons were cloned into pMD18-T vectors (Invitrogen) and transformed into *Escherichia coli* TOP10 (Invitrogen). Transformants were plated onto LB agar plates containing ampicillin (50 µg/ml), isopropyl β-D-1-thiogalactopyranoside (IPTG) (20% w/v), and X-gal solution (2% w/v). After incubation at 37°C overnight, positive transformants were selected, stored in 96-well microtiter plates, and further inspected of inserts by PCR amplification with the universal primers M13F (5′-GTAAAACGACGGCCAG-3′) and M13R (5′-CAGGAAACAGCTATGAC-3′). A total of 768 verified positive transformants were subjected to BigDye Terminator (Applied Biosystems) and analyzed on ABI 3730×l sequencers (Applied Biosystems). Inspection of sequence chromatograms was performed with Sequence Scanner v1.0 (https://products.appliedbiosystems.com/). Trimming and sequence assembling were performed with CodonCode Aligner (http://www.codoncode.com/). Sequence alignment and chimera-check were performed with the NAST aligner [29] and Bellerophon [35], which excluded five possible chimeras and obtained a total of 623 qualified near full-length 16S rRNA genes. The alignment was further imported into the ARB package [30], with which phylogenetic positions of these sequences were determined. OTU determination and species richness estimation were performed by DOTUR [36] with 97% sequence identity.

### Nucleotide Accession Numbers

The CoII gene of the present termite was deposited at GenBank under the accession number JN223393. The 16S rRNA gene sequences were deposited under the accession numbers JN619456-JN620078. The raw pyrosequencing data of the fungus-cultivating termite gut metagenome were deposited at NCBI Sequence Read Archive (SRA) under the accession number SRA045578.

## Results and Discussion

### Metagenomic Overview of the Gut Microbiota of a Fungus-cultivating Termite

To investigate the symbiotic roles of the gut microbiota in fungus-cultivating termites, we performed a metagenomic inventory of that of *O. yunnanensis* by examining both the phylogeny and metabolic potentials. Through pyrosequencing a total of 548,807 reads averaging 234 base pairs (Table 1 and Figure S2 in File S1) were obtained. Assembly of the raw sequences was tried but failed. So, unassembled dataset was subjected to further analysis. It showed that 68.25% reads could be assigned by MEGAN, among which 96.3% were affiliated to bacteria, and only 3.26% were affiliated to eukaryotes (Table 1), including 1229 reads for Arthropoda and 246 for Basidiomycota (data not shown). This further testified the effectiveness of the eukaryote-excluding pretreatment we have developed [14] and applied. Environmental gene tags (EGTs) are short sequences from the metagenome that contain fragments of functional genes. Relative abundance of functional genes reflects its relative importance to the habitat [37]. Here, metagenomic reads annotated by SEED subsystems were defined as EGTs and approximately 30% of all obtained reads (Table 1) could be classified as EGTs which could be used for further gene-centric comparative analysis.

**Table 1:** Summary of pyrosequencing data from the fungus-cultivating termite whole gut metagenome.

Parameters	Values
No. of reads	548,807
Total length of reads, bp	128,474,271
Average length of reads, bp	234
All reads assigned by MEGAN* (% of all reads)	374,549 (68.25)
Archaea (% of all binned reads)	1,062 (0.28)
Bacteria (% of all binned reads)	360,831 (96.3)
Eukarya (% of all binned reads)	12,216 (3.26)
Virus (% of all binned reads)	440 (0.12)
Reads with nr hits† (% of all reads)	314,096 (57.23)
Reads assigned to SEED subsystems (EGTs)§ (% of all reads)	163,902 (29.87)
Reads with 16S rRNA gene hits¶ (% of all reads)	539 (0.098)

* Taxonomy assigned by MEGAN based on reads with nr hits (E≤10^−2^).

† The E-value cutoff for BLASTX search against the NCBI nr database is 10^−5^.

§ The E-value cutoff for BLASTX search against SEED databases was 10^−5^. Metagenomic reads annotated by SEED subsystems were defined as EGTs.

¶ The E-value cutoff for BLASTN searches against the RDP database for 16S rRNA genes was 10^−5^ with a minimum length of 100 bp and a minimum sequence identity of 75%.

### Dominance of Bacteroidales and Clostridiales in the Gut Microbial Community

The community structure of the given metagenome was explored both by MEGAN binning all coding sequences in the metagenomic dataset [27] and by investigating the 16S rRNA genes, including those directly derived from the sequenced metagenome and those from a PCR-based clone library. And the results of each approach largely correlated well with one other. Analysis of the 623 qualified near full-length PCR-amplified bacterial 16S rRNA gene sequences revealed a broadly diverse community comprised of 11 phyla and 187 phylotypes (with 97% sequence identity threshold) (Table 2 and Table S1 in File S1). Nonparametric estimators ACE and Chao1 suggested that nearly 84.4% of the total species diversity of the gut microflora had been presented at the given phylotype threshold (Figure S3 in File S1), indicating a relatively sufficient sampling depth of the clone library compared to one of our previous attempts [13]. The degree of saturation decreased progressively as the threshold increased (Figure S3 in File S1). Of the 187 phylotypes, 80.7% proved to be novel, showing similarities lower than 97% with their best hits (Table S1 in File S1), implying a unique set of gut microflora in *O. yunnanensis*. And 130 phylotypes (representing 501 of the 623 sequences) had best hits from termite-derived clones, including termite genera from all feeding guilds, involving *Odontotermes, Macrotermes, Nasutitermes, Microcerotermes, Cubitermes, Coptotermes,* and *Reticulitermes* etc (Table S1 in File S1). While 62.3% of the 130 phylotypes (representing 80.6% of the 501 sequences) had best hits from fungus-growers, including *O. formosanus* [12], *Macrotermes. gilvus* [5], and *Microcerotermes. michaelseni* [11] (Table S1 in File S1).

**Table 2:** Comparison of community composition revealed by 16S rRNA genes and all environmental gene tags.

Phylum†	16S rRNA genes from metagenome (fragment)		16S rRNA genes from clonelibrary (full-length)		All coding sequences*	
Bacteroidetes	231	(42.86%)	302	(48.48%)	127904	(48.65%)
Firmicutes	134	(24.86%)	165	(26.48%)	57110	(21.72%)
Proteobacteria	64	(11.87%)	49	(7.87%)	38259	(14.55%)
Spirochaetes	44	(8.16%)	39	(6.26%)	8785	(3.34%)
Synergistetes	17	(3.15%)	28	(4.49%)	4540	(1.73%)
Planctomycetes	25	(4.64%)	12	(1.93%)	4914	(1.87%)
Elusimicrobia	0	(0%)	10	(1.61%)	2341	(0.89%)
Actinobacteria	8	(1.48%)	6	(0.96%)	4935	(1.88%)
Chlorobi	8	(0.74%)	6	(0.96%)	1911	(0.73%)
Candidate division TM7	3	(0.56%)	5	(0.80%)	0	(0%)
Deferribacteres	0	(0%)	1	(0.16%)	235	(0.09%)
Caldiserica	4	(0.74%)	0	(0%)	0	(0%)
Lentisphaerae	1	(0.19%)	0	(0%)	432	(0.16%)
Chloroflexi	1	(0.19%)	0	(0%)	332	(0.13%)
Fibrobacteres	1	(0.19%)	0	(0%)	94	(0.04%)
Candidate division OP11	2	(0.37%)	0	(0%)	0	(0%)
Acidobacteria	0	(0%)	0	(0%)	375	(0.14%)
Total	539	(100%)	623	(100%)	262881	(95.92%)

† Phyla were sorted by the abundance of full-length 16S rRNA sequences.

* All coding sequences is identified by BLASTX search against the NCBI nr database (E≤10^−5^).

Other phyla with low abundances of coding sequences are not listed here.

Of the 11 phyla, Bacteroidetes proved to be predominant (50 phylotypes, 302 sequences) (Table 2). Among these, members of the genus *Alistipes* were especially prevalent (23 phylotypes, 171 sequences) (Table S1 in File S1), including two most frequently detected phylotypes (with 49 and 51 sequences respectively). The second most abundant phylum proved to be Firmicutes, comprising even more phylotypes but with fewer sequences (71 phylotypes, 165 sequences) (Table 2), among which 68 phylotypes belonged to the order Clostridiales (Table S1 in File S1). Proteobacteria ranked third in the community (22 phylotypes, 49 sequences) and was followed by Spirochaetes (15 phylotypes, 39 sequences) (Table 2 and Table S1 in File S1). It is noticeably that the phylotype-based phylum composition revealed here largely resembles that observed in our previous investigation of the same termite species, which used a direct method for DNA extraction (QIAamp DNA Stool Mini Kit) and achieved only 83 available sequences (unsubmitted) and an obviously unsaturated rarefaction curve [13]. MEGAN binning of all predicted coding sequences as well as 539 qualified 16S rRNA gene fragments also revealed a community composition generally coincided with that deduced from the 16S rRNA gene clone library at the phylum level (Table 2), indicating a relatively random sampling of the sequenced library. On the whole, resembling other investigations of community profiles of fungus-growers based on molecular methods [5], [12], the present *O. yunnanensis* exhibited a gut microbiota dominated by termite-specific clades of as-yet-uncultured Bacteroidales and Clostridiales, which is in obvious contrast to the predominance of Spirochaetes in the investigated wood-feeders [15], [38], [39], especially the paunch compartments [39]. Actually, this remarkable community structural differences have led to apparent metabolic divergences in SEED subsystem-based functional categories, that subsystems of motility and chemotaxis (Figure S4 in File S1), specifically, bacterial chemotaxis and flagellar motility in prokaryota (Figure S5A in File S1) were significantly overrepresented in the highly mobile Spirochaetes-dominated *Nasutitermes* hindgut metagenome [15]. While Bacteroidales conjugative transposons were obviously overrepresented in the Bacteroidetes-dominated fungus-grower gut microbiome (2200 EGTs, Figure S5B in File S1), suggesting that they may aid in the adaption of this diverse group of bacteria to the gut environments by means of DNA transfer between Bacteroides and other bacteria residing there, as suggested in the human gut [40].

### Potential of Gut Microbiota for Hosts’ Plant Matter Degradation

**Figure 1:**
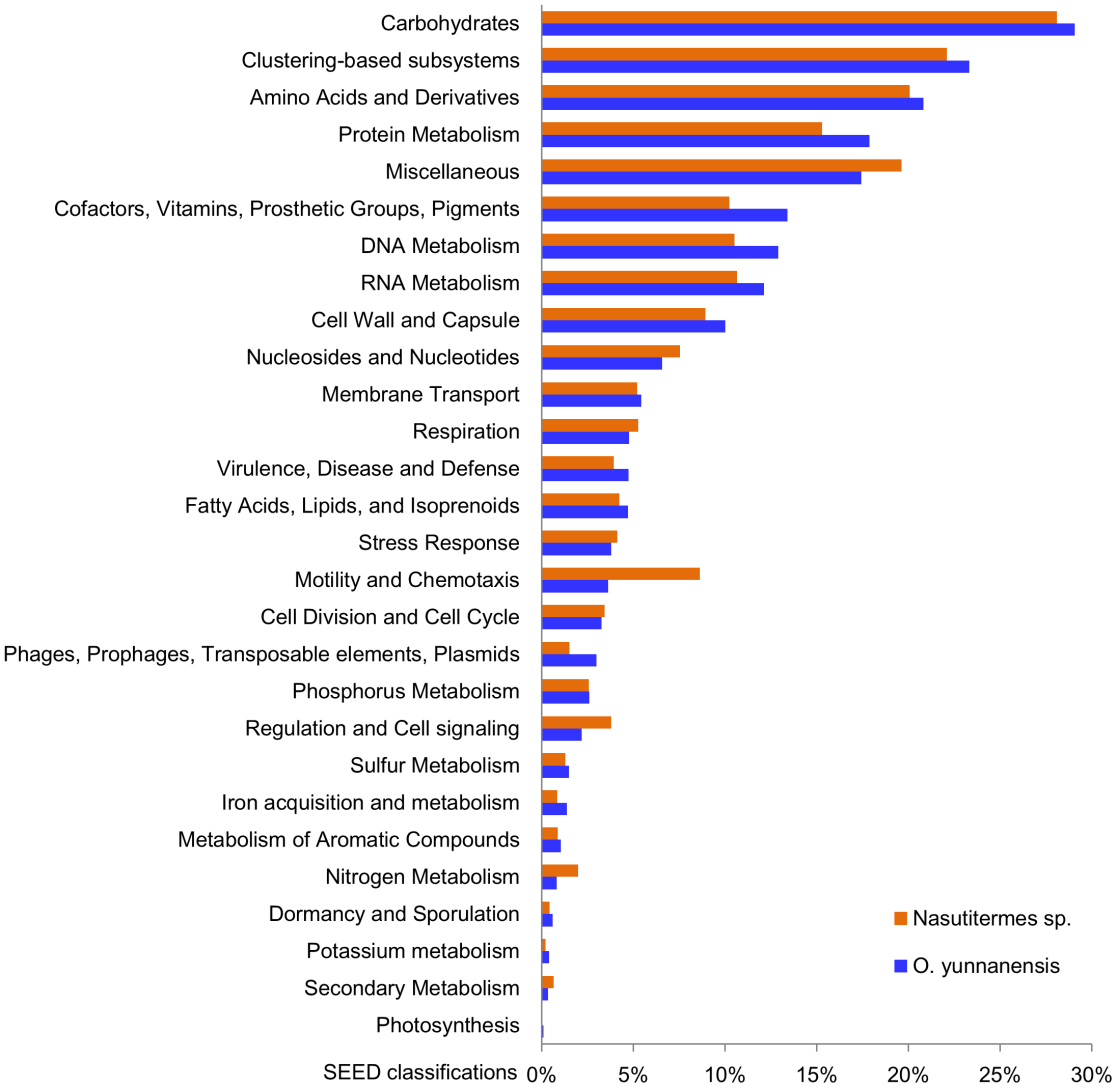
SEED profiles of gut microbiota of fungus-cultivating *O. yunnanensis* and wood-feeding *Nasutitermes* sp. [15]. The proportions of environmental gene tags (EGTs) in each classification with respect to the number of all annotated ones in individual metagenome were presented.

Fungus-cultivating termite symbiotic systems have long been recognized for their enormous influence on the decomposition of plant matters in tropical and subtropical ecosystems. However, how lignocelluloses are degraded within this system remains obscure. One aim of this study was to explore the roles the gut microbiome of fungus-cultivating termites may have played in this process. Thus all carbohydrate-active enzymes (CAZymes) harbored by this microbiome were predicted and classified into families based on the sequence similarities of their catalytic or carbohydrate-binding modules, as according to the principle of the carbohydrate-active enzymes database (CAZy) for CAZy family classification [41]. Indeed, a broad array of genes or gene modules distributing throughout diverse CAZy families that are responsible for lignocellulose degradation were detected (Table 3 and Table S2 in File S1), which could also be reflected by the highest proportion of functional genes categorized into the SEED carbohydrate subsystems (Figure 1), indicating a potential role of gut microbes for host’s plant matter digestion in addition to the symbiotic fungi. Concretely, the largest proportion of the identified CAZymes were most similar to debranching and oligosaccharide-degrading enzymes (3177 CAZymes modules, 43.9%), while relatively few were assigned to cellulases and endohemicellulases (205 CAZymes modules, 2.8%) (Table 3), indicating a more obvious potential of gut microbiota in processing plant oligomers of short or sides chains for the termite host. When compared the CAZyme profile with that of a few other resolved lignocellulose-degrading communities such as the wood-feeding termite hindgut [15], leaf cutter ant fungus gardens [18] and the tammar wallaby foregut [17], it showed that the lignocelluloytic potential of the present metagenome was quite divergent from that of the higher wood-feeding *Nasutitermes sp.*, and was more similar with those of the leaf cutter ant fungus garden and wallaby gut, both containing relatively fewer cellulases and endohemicellulases but more abundant debranching and oligosaccharide-degrading enzymes (Table S4 in File S1).

**Table 3:** Selected carbohydrate-active gene modules detected in the fungus-cultivating termite gut metagenome.

CAZy family*	Known CAZy Activities§	Reads in *O. yunnanensis*
	**Cellulases and endohemicellulases**	
GH5	Cellulase, β-mannnanse, β-1,3-glucosidase, β-1,4-endoxylanase, others	67
GH8	Cellulase, endo-1,4-β-xylanase, chitosanase, others	7
GH9	Endoglucanase, cellobiohydrolase, β-glucosidase	59
GH10	Xylanase, β-1,3-endoxylanase	29
GH26	β-1,3-Xylanase, mannanase	14
GH74	Endoglucanase; oligoxyloglucan reducing end cellobiohydrolase; xyloglucanase	23
GH113	β-mannanase	2
GH124	Endoglucanase	4
Total		205 (2.8%)
	**Debranching enzymes and Oligosaccharide degrading enzymes**	
GH1	β-Glucosidase, β-galactosidase, β-mannosidase	59
GH2	β-Galactosidase, β-mannosidase, β-glucuronidase	742
GH3	β-Glucosidase, β-xylosidase, α-L-arabinofuranosidase, others	183
GH4	α-Glucosidase, α-galactosidase, α-glucuronidase	35
GH27	α-Galactosidase	10
GH29	α-L-fucosidase	145
GH30	Glucosylceramidase, β-1,6-glucanase, β-xylosidase, β-glucosidase	67
GH31	α-Glucosidase, α-xylosidase	122
GH35	β-Galactosidase	20
GH36	α-Galactosidase	135
GH39	β-Xylosidase, α-L-iduronidase	23
GH42	β-Galactosidase	54
GH43	Xylanase, β-xylosidase, α-Larabinofuranosidase, arabinanase	282
GH51	Endoglucanase, α-L-arabinofuranosidase	127
GH53	β-1,4-endogalactanase	5
GH67	α-Glucuronidase	36
GH78	α-L-rhamnosidase	225
GH93	Exo-1,5-α-L-arabinanase	9
GH94	Cellobiose phosphorylase, chitobiose phosphorylase	60
GH97	α-Glucosidase, α-galactosidase	162
GH106	α-L-Rhamnosidase	161
GH115	Xylan α-1,2-glucuronidase, α-(4-O-methyl)-glucuronidase	31
GH116	Acid β-glucosidase, β-glucosidase, β-xylosidase	3
GH120	β-Xylosidase	2
GH121	β-L-arabinobiosidase	5
GH127	β-L-arabinofuranosidase	143
CE1	Acetyl xylan esterase, feruloyl esterase, carboxylesterase	223
CE2	Acetyl xylan esterase	6
CE3	Acetyl xylan esterase	26
CE5	Acetyl xylan esterase; cutinase	1
CE6	Acetyl xylan esterase	17
CE7	Acetyl xylan esterase, cephalosporin-C deacetylase	58
Total		3177 (43.9%)
	**Fungal cell wall degrading enzymes**	
GH18	Chitinase, endo-b-N-acetylglucosaminidase, non-catalytic proteins	54
GH19	Chitinase	6
GH20	β-Hexosaminidase, lacto-N-biosidase	186
GH38	α-Mannosidase, mannosyl-oligosaccharide α-1,3-1,6-mannosidase and α-1,3-mannosidase	30
GH55	Exo-1,3-glucanase, endo-1,3-glucanase	43
GH76	α-1,6-Mannanase	22
GH81	Endo-β-1,3-glucanase	7
GH85	Endo-β-N-acetylglucosaminidase	2
GH92	Mannosyl-oligosaccharide α-1,2-mannosidase; α-1,3-mannosidase and α-1,6-mannosidase; α-1,2-mannosidase; α-1,3-mannosidase, others	427
GH99	Glycoprotein endo-α-1,2-mannosidase	19
GH125	Exo-α-1,6-mannosidase	56
GH128	β-1,3-glucanase	1
CE4	Chitin, chitooligosaccharide, peptidoglycan GlcNAc and MurNAc deacetylase	81
Total		934 (12.9%)

* The carbohydrate-active enzymes database (CAZy), http://www.CAZy.org.

§ Known CAZy activities were given according to the CAZy database.

† Carbohydrate-active enzymes were detected with the set of CAZy family-specific HMMs defined by Yin et al [24], as deposited on dbCAN (http://csbl.bmb.uga.edu/dbCAN/), in the BLAST searches (E≤10^−4^). All identified CAZymes were listed in Table S2. Besides, searches for GHs-associated domains were also performed and indexed in Table S3.

This digestive profile could be interpreted by several factors, which are not mutually exclusive. One may be that, compared to the pure wood consumed by *Nasutitermes,* leaves and grasses were also considerably foraged by fungus-growers, a diet resembling that of leaf cutter ants and wallaby. Secondly, a potential complementary or synergistic cooperation of the gut microbiota with the ectosymbiotic fungi may be applied here. This assumption was supported to some extent both by the results of a subtractive EST analysis of the symbiotic fungi of *Macrotermes gilvus* [42] and the results of our previous functional exploration of the gut metagenome of *Macrotermes annandalei* [14]. That in *M. gilvus*, a total of 70 ESTs (29 unique transcripts after assembly) relevant to plant cell wall degradation were identified, while among these, 60 ESTs (21 tentative consensus sequences) were responsible for degrading backbones of plant cellulose, hemicelluloses, and pectins [42]. While in *M. annandalei*, beta-glucosidases were preferentially recovered by functional screening from the fosmid library of its gut metagenome [14]. However, considering potential differences between *Odonterterms* and *Macrotermes,* more investigations of *Odonterterms* species themselves remains, to further elucidate this cooperative pattern. Thirdly, since a microbiome associated with the fungus garden also exists, as indicated by our previous investigations [13], the respective metagenome may also play a role in plant biomass decomposition, as the case revealed in the leaf cutter ant gardens [18], [19].

**Figure 2:**
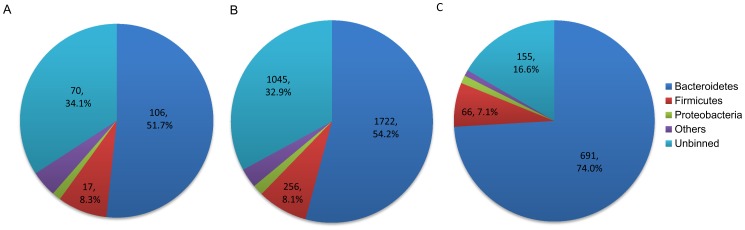
Taxonomic assignment of metagenomic fragments associated with plant and fungal cell wall degradation. Taxonomic assignment of CAZymes associated with degradation of main chains of plant cell wall polysaccharides (A), short or side chains thereof (B), and fungal cell wall saccharides (C).

Taxonomic assignment of CAZymes relevant to plant cellulose and hemicellulose degradation revealed that, both backbone-processing enzymes and debranching or oligomer-processing enzymes were mainly assigned to Bacteroidetes (51.7% and 54.2%, respectively), followed by Firmicutes (8.3% and 8.1%, respectively) (Figure 2A and Figure 2B). This indicated that the dominant groups of Bacteroidetes and Firmicutes in the given fungus-cultivating termite gut ecosystem should also be the major contributors of the plant matters-degrading enzymes there, in contrast to the case in the *Nasutitermes* termite, where the predominant Spirochaetes and Fibrobacteres are the main contributors [15].

As is the case in the wood-feeding *Nasutitermes* [15], the present metagenome harbored no typical oxidative lignin-degrading enzymes, (lignin peroxidase EC 1.11.1.14, manganese peroxidase EC 1.11.1.13, and laccase EC 1.10.3.2). However, it did show more obvious metabolic potential of utilizing aromatic compounds or central aromatic intermediates than that of the *Nasutitermes* metagenome according to our SEED subsystem-based comparative analysis of both metagenomes. Specifically, subsystems: anaerobic benzoate metabolism, gentisate degradation, homogentisate pathway of aromatic compound degradation, central meta-cleavage pathway of aromatic compound degradation, catechol branch of beta-ketoadipate pathway, and the salicylate and gentisate catabolism pathway, were selectively enriched in the present dataset (Figure S5C and Table S5 in File S1). As lignin is a well known plant polymer composed of aromatic structural units, and lignin decomposition on the fungus combs has already been shown in many *Macrotermes* species [43], [44], [45] or indicated in some *Odontotermes* species [45], [46], this metabolic capacity of fungus-cultivating termite gut microbiota could possibly be used for assimilating lignin-derived central aromatic intermediates as growth substrates during the second gut passage. Actually, mineralization of benzoate, a most common intermediate in anaerobic metabolism of aromatic compounds, has already been detected during gut passage in the cases of two fungus-cultivators [47], [48].

### Metabolic Adaption to a Fungal Diet of Gut Microbiome in Fungus-cultivating Termites

In addition to the genetic potential for plant matter degradation, a diverse variety of gene modules (934 CAZymes modules, 12.9%) (Table 3) encoding enzymes that process typical fungal cell wall components [49], [50], [51], [52], [53], including chitin, beta-1,3-glucan, and N- and O-linked oligosaccharide moieties in the mannan or galactomannan glycoproteins, were also detected in the present metagenome. These enzymes included chitinases, glucanases and mannosidases [54], [55], [56] that distributed across GH families 18, 19, 20, 38, 55, 76, 81, 85, 92, 99, 125, 128, CE family 4 (Table 3) and also CBM families 5, 12, 14, 33, 54 and 56 (Table S2 in File S1), as seen from the carbohydrate-active enzymes database (http://www.cazy.org/). Indeed, chitinolytic activity has already been detected in the gut homogenate of two fungus-growers [44]. Taxonomic binning of gene modules affiliated to CAZy families responsible for fungal cell wall decomposition showed that, they should also be contributed mainly by the dominant groups of Bacteroidetes (74.0%) and Firmicutes (7.1%) (Figure 2C).

Parallel to the discovery of a diverse variety of gene modules associated with fungal cell wall degradation, another apparent metabolic divergence between the wood-feeding and fungus-cultivating termite gut microbiomes driven by this obvious dietary difference could also be reflected in the gene-centric SEED comparisons, that most subsystems of nitrogen metabolism, including ammonia assimilation, nitrate and nitrite ammonification, and especially nitrogen fixation, were significantly underrepresented in the sequenced metagenome compared to that of the *Nasutitermes* P3 metagenome (Figure S4, Figure S5D and Table S6 in File S1). Indeed, our attempt to amplify the *nifH* gene with the primer designed by Kirshtein et al [57] from this termite species failed, in contrast to both the case in a wood-feeding *Globitermes* species we examined (Figure S6 in File S1) and the rich diversity of near-full length *nifH* homologues reported in the wood-feeding *Nasutitermes* species [15], as also could be seen in our SEED annotations (Table S6 in File S1). These observations are congruent with the unique dietary behavior of fungus-cultivating termites that fungus is elaborately deposited, cultivated, and harvested by worker termites and the fact that fungal mycelium itself is a nitrogen-rich food source [58], [59] which could be dissimilated and utilized by gut microbes to partially compensate the nitrogen-depleted lignocellulosic diet for themselves as well as termite hosts.

### Supplement to Symbiotic Hypothesis on Fungus-cultivating Termites

We presented here a metagenomic inventory of the gut microbiome of a fungus-cultivating termite, from which we unveiled their genetic potential for digesting both the dietary lignocelluloses and fungus biomass for termite hosts. Comparison of the data with that of a previously sequenced wood-feeding *Nasutitermes* species revealed both distinct microbial community compositions and divergent metabolic potentials. Although these divergences could not be excluded from the possible implications that in the *Nasutitermes* species, only microflora within the predominant microbe-harboring P3 segment was included, while minor members associated with the paunch epithelium and those may present in other segments [39] were excluded compared to the whole gut microbiota included in the present study, they are more likely to be attributed to the substantially different feeding habits of the termite hosts. Firstly, the gut microbiome of fungus-cultivating termites showed obvious potential to participate in host’s plant fiber digestion, just that it tended to play a more important role in processing side chains and oligomers. This pyrosequencing-based inventory together with our previous function-based screening [14] have fundamentally updated our present knowledge of the potential digestive roles of gut microbes for Macrotermitinae. Meanwhile, subsystems relevant to metabolism of central aromatic intermediates and compounds were selectively overrepresented here, suggesting that gut microflora of fungus-cultivating termites could potentially utilize aromatic compounds, most probably lignin-derived, as carbon or energy source. Secondly, the sequenced microbiome exhibited an apparent metabolic potential of dissimilating fungal cell walls, including both chitins and sugar chains of glycoproteins thereof, while spared a economical strategy of nitrogen fixation. Based on these, as well as existing understandings of the symbiotic mechanisms underlying this efficient lignocellulose-biorecycling system (the following a to c), we brought instructive insights into how gut microbiome of fungus-cultivating termite may help the symbiotic system work as a whole (the following d): (a) Plant matters are first collected and carried by old workers to the nest. (b) Young workers mechanically masticate these foodstuffs, as a way of pretreatmemt, and ingest fungus nodules to produce inoculated primary feces as fresh fungus comb. (c) As the *Termitomyces* fungi grow thereon, degradation of plant cell wall components proceeds by fungal enzymes, forming mature combs comprising both partially processed plant matters and senescent mycelia. (d) Old workers consume mature combs to further degrade oligosaccharides thereof, metabolize central aromatic compounds, and assimilate fungal biomass as nutrient supplement, for the termite hosts.

Given that the symbiotic pattern of Macrotermitinae termites with *Termitomyces* may vary among termite taxa [4], [44], [60], more direct studies targeting *Odontotermes* themselves, including composition of the original forages, compositional transformations of fungus combs as they mature, hydrolytic potential of microbiome associated with the fungus gardens, and to further extent, a direct transcriptomic analysis of the given symbiotic *Termitomyces* species, remain to be established to further elucidate this peculiar symbiosis, which should serve as an informative representative for understanding the nutritional basis underlying fungus-cultivating termite symbiotic systems.

## Supporting Information

File S1 **Figure S1. DGGE fingerprint of the V3 region of 16S rRNA genes amplified from termite gut eDNA prepared with two different fractionation procedures.** V3 region of 16S rRNA genes was amplified with primers Eubac7 Vf-GC/Vr from 10 ng of eDNA prepared by 1: directly homogenizing whole gut tissues with a 2-ml Tenbroeck tissue grinder, and centrifugate at 200×g to remove coarse particles; 2: homogenizing with pipette after trypsin digestion, and centrifugating at 800×g to extrude eukaryotic cells. Detailed procedures for DGGE could be seen in our previous work of preparing gut eDNA for *M. annandalei* [14]. **Figure S2. Distribution of 454 sequences of the whole gut metagenome of** ***O. yunnanensis*** **. Figure S3. Collectors’ curves (Collector, Chao1, and ACE) derived from full-length 16S RNA gene library of** ***O. yunnanensis*** **whole gut metagenome.** Phylotype cutoffs were 97%, 98%, and 99%, respectively. **Figure S4. Statistically different SEED classifications between the gut microbiomes of** ***O. yunnanensis*** **and the** ***Nasutitermes*** **sp.** [**15**] **.** Classifications statistically overrepresented in the *Odontotermes* metagenome were marked with blue circles, while those statistically overrepresented in the *Nasutitermes* metagenome were marked with orange ones (P<0.05 and Ratio of proportions >1.1 were shown). SEED subsystems-based annotation of both metagenomes was performed as described in the methods. The proportions of environmental gene tags (EGTs) in each classification with respect to the total number of SEED annotated ones in individual metagenome were calculated, based on which ratio of proportions of each classification in the two datasets were further calculated. Gene-centric statistic analysis was performed with two-sided Fisher’s exact test implemented in the STAMP program [33]. P values were corrected by the Benjamini-Hochberg multiple test and confidence intervals were calculated by the Asymptotic method. **Figure S5. Subsystem distributions in partial statistically different SEED classifications between the gut microbiomes of** ***O. yunnanensis*** **and the** ***Nasutitermes*** **sp.** [**15**] Subsystem distribution of motility and chemotaxis (A), phages, prophages, transposable elements, plasmids (B), metabolism of aromatic compounds (C), and nitrogen metabolism (D). Noticeably, in (C) the two subsystems statistically overrepresented in the *Nasutitermes* metagenome both belong to peripheral pathways of catabolism of aromatic compounds (labeled in red caption), while all subsystems statistically enriched in the *Odontotermes* metagenome belong to metabolism of central aromatic intermediates or aromatic compounds (labeled in black caption, also see Table S5 in File S1). Statistic analysis was performed with the same procedures and parameters as for Figure S4. **Figure S6. Electrophoresis detection of the PCR amplification product of the** ***nifH*** **Gene.** The highly degenerate primers (Pf: 5′-TGXGAXCCYAAZGCYGA-3′ X = T or C, Y = A,C,G, or T and Pr: 5′-AWYGCCATCATXTCYCC-3′ Z = A or G, W = A,T, or G) designed by Kirshtein et al [57]. were used to amplify the ∼360 bp fragment of the *nifH* gene, which encodes the iron protein of nitrogenase that catalyzes N_2_ fixation. M, Quick-Load 100 bp DNA Ladder; 1, amplification products from the whole gut metagenomic DNA of the higher wood-feeding *Globitermes brachycerastes*; 2, amplification products from the whole gut metagenomic DNA of the fungus-cultivating *O. yunnanensis*; 3, amplification products from the whole gut metagenomic DNA of the fungus-cultivating *Macrotermes annandelei.* It revealed that the ∼360 bp fragment of the *nifH* gene which encodes the iron protein of nitrogenase could only be amplified from the wood-feeding higher termite species. **Table S1. Phylotype representatives of 16S rRNA sequences obtained from clone library in the whole gut microbiome of** ***Odontotermes yunnanensis*** **. Table S2. Carbohydrate-active gene modules detected in the gut metagenome of** ***O.yunnanensis*** **. Table S3. Domains often associated with GH catalytic domains detected in the gut metagenome of** ***O.yunnanensis*** **. Table S4. Comparison of CAZy profiles of the whole gut metagenome of** ***O.yunnanensis*** **with those of the leaf cutter ant fungus garden** [**18**] **, wallaby foregut** [**17**] **, and the wood-feeding** ***Nasutitermes*** **sp. hindgut** [**15**] **. Table S5. Distribution of statistically different subsystems in metabolism of aromatic compounds between the gut microbiomes of** ***O. yunnanensis*** **and the** ***Nasutitermes*** **sp. Table S6. Composition of statistically different subsystems in nitrogen metabolism between the gut microbiomes of** ***O. yunnanensis*** **and the** ***Nasutitermes*** **sp.**
(DOC)
