# Supplementary material for: Metagenomic Insights into Metabolic Capacities of the Gut Microbiota in a Fungus-Cultivating Termite (Odontotermes yunnanensis)
Source: PLoS One. 2013 Jul 17;8(7):e69184. doi: 10.1371/journal.pone.0069184 (PMC3714238; doi:10.1371/journal.pone.0069184)
Supplement: File S1 — Figure S1. DGGE fingerprint of the V3 region of 16S rRNA genes amplified from termite gut eDNA prepared with two different fractionation procedures. V3 region of 16S rRNA genes was amplified with primers Eubac7 Vf-GC/Vr from 10 ng of eDNA prepared by 1: directly homogenizing whole gut tissues with a 2-ml Tenbroeck tissue grinder, and centrifugate at 200×g to remove coarse particles; 2: homogenizing with pipette after trypsin digestion, and centrifugating at 800×g to extrude eukaryotic cells. Detailed procedures for DGGE could be seen in our previous work of preparing gut eDNA for M. annandalei [14]. Figure S2. Distribution of 454 sequences of the whole gut metagenome of O. yunnanensis . Figure S3. Collectors’ curves (Collector, Chao1, and ACE) derived from full-length 16S RNA gene library of O. yunnanensis whole gut metagenome. Phylotype cutoffs were 97%, 98%, and 99%, respectively. Figure S4. Statistically different SEED classifications between the gut microbiomes of O. yunnanensis and the Nasutitermes sp. [15] . Classifications statistically overrepresented in the Odontotermes metagenome were marked with blue circles, while those statistically overrepresented in the Nasutitermes metagenome were marked with orange ones (P<0.05 and Ratio of proportions >1.1 were shown). SEED subsystems-based annotation of both metagenomes was performed as described in the methods. The proportions of environmental gene tags (EGTs) in each classification with respect to the total number of SEED annotated ones in individual metagenome were calculated, based on which ratio of proportions of each classification in the two datasets were further calculated. Gene-centric statistic analysis was performed with two-sided Fisher’s exact test implemented in the STAMP program [33]. P values were corrected by the Benjamini-Hochberg multiple test and confidence intervals were calculated by the Asymptotic method. Figure S5. Subsystem distributions in partial statistically different SEED classificati [file pone.0069184.s001.doc]

**Metagenomic insights into metabolic capacities of the gut microbiota in a fungus-cultivating termite (*Odontotermes yunnanensis*)**

| **Content of Supporting Information** | |
| --- | --- |
| Figure S1. DGGE fingerprint of the V3 region of 16S rRNA genes amplified from termite gut eDNA prepared by two fractionation procedures. | P2 |
| Figure S2. Distribution of 454 sequences of the whole gut metagenome of *O. yunnanensis.* | P3 |
| Figure S3. Collectors’ curves (Collector, Chao1, and ACE) derived from full-length 16S RNA gene library of *O. yunnanensis* whole gut metagenome. | P4 |
| Figure S4. Statistically different SEED classifications between the gut microbiomes of *O. yunnanensis* and the *Nasutitermes* sp.. | P5 |
| Figure S5. Subsystem distributions in partial statistically different SEED classifications between the gut microbiomes of *O. yunnanensis* and the *Nasutitermes* sp.. | P6 |
| Figure S6. Electrophoresis detection of the PCR amplification product of the *nifH* Gene. | P7 |
| Table S1. Phylotype representatives of 16S rRNA sequences obtained from clone library in the whole gut microbiome of *Odontotermes yunnanensis.* | P8 |
| Table S2. Carbohydrate-active gene modules detected in the gut metagenome of *O.yunnanensis.* | P13 |
| Table S3. Domains often associated with GH catalytic domains detected in the gut metagenome of *O.yunnanensis.* | P17 |
| Table S4. Comparison of CAZy profiles of the whole gut metagenome of *O.yunnanensis* with those of the leaf cutter ant fungus garden [18], wallaby foregut [17], and the wood-feeding *Nasutitermes* sp. hindgut [15]. | P19 |
| Table S5. Distribution of statistically different subsystems in metabolism of aromatic compounds between the gut microbiomes of *O. yunnanensis* and the *Nasutitermes* sp.*.* | P22 |
| Table S6. Composition of statistically different subsystems in nitrogen metabolism between the gut microbiomes of *O. yunnanensis* and the *Nasutitermes* sp.. | P23 |


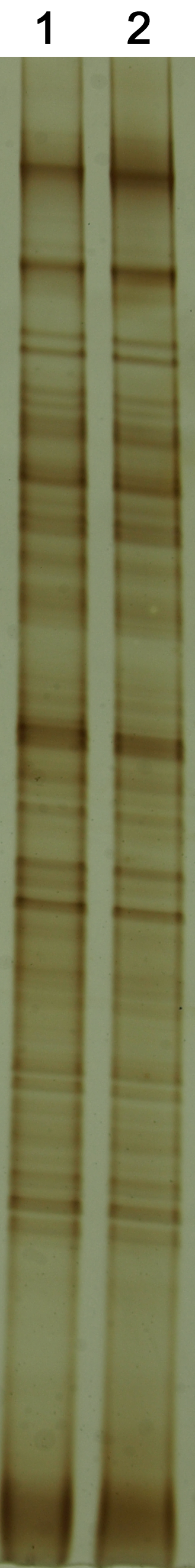


**Figure S1. DGGE fingerprint of the V3 region of 16S rRNA genes amplified from termite gut eDNA prepared with two different fractionation procedures.** V3 region of 16S rRNA genes was amplified with primers Eubac7 Vf-GC/Vr from 10 ng of eDNA prepared by 1: directly homogenizing whole gut tissues with a 2-ml Tenbroeck tissue grinder, and centrifugate at 200 x g to remove coarse particles; 2: homogenizing with pipette after trypsin digestion, and centrifugating at 800 x g to extrude eukaryotic cells. Detailed procedures for DGGE could be seen in our previous work of preparing gut eDNA for *M. annandalei* [14].

**
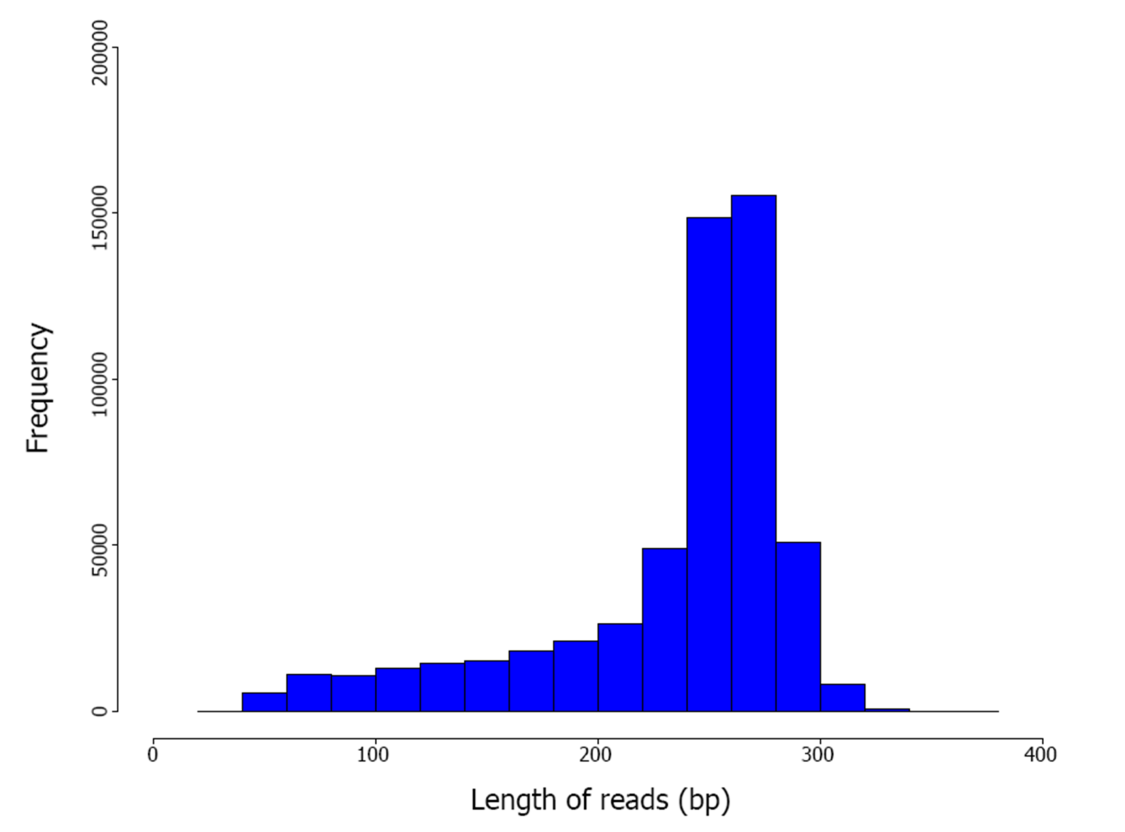
**

**Figure S2.** **Distribution of 454 sequences of the whole gut metagenome of *Odontotermes yunnanensis*.**


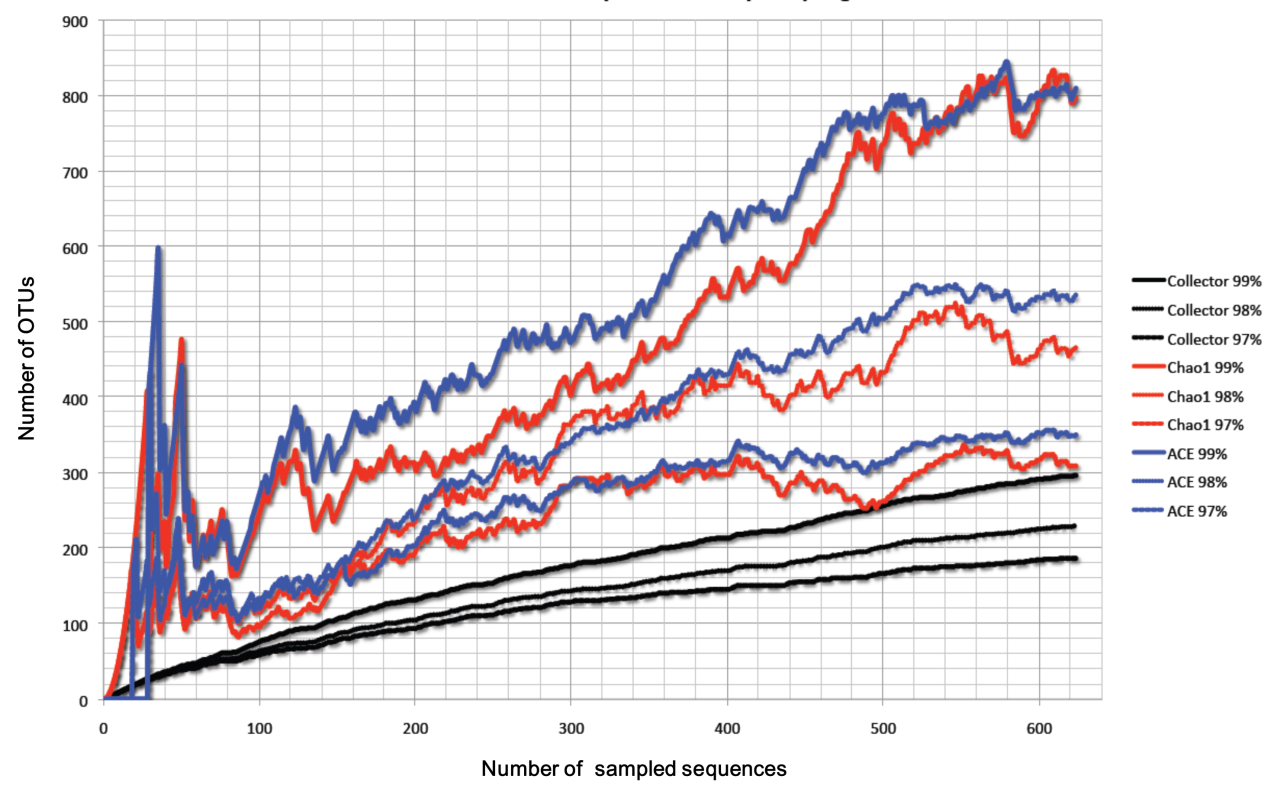


**Figure S3.** **Collectors’ curves (Collector, Chao1, and ACE) derived from full-length 16S RNA gene library of *O. yunnanensis* whole gut metagenome.** Phylotype cutoffs were 97%, 98%, and 99%, respectively.


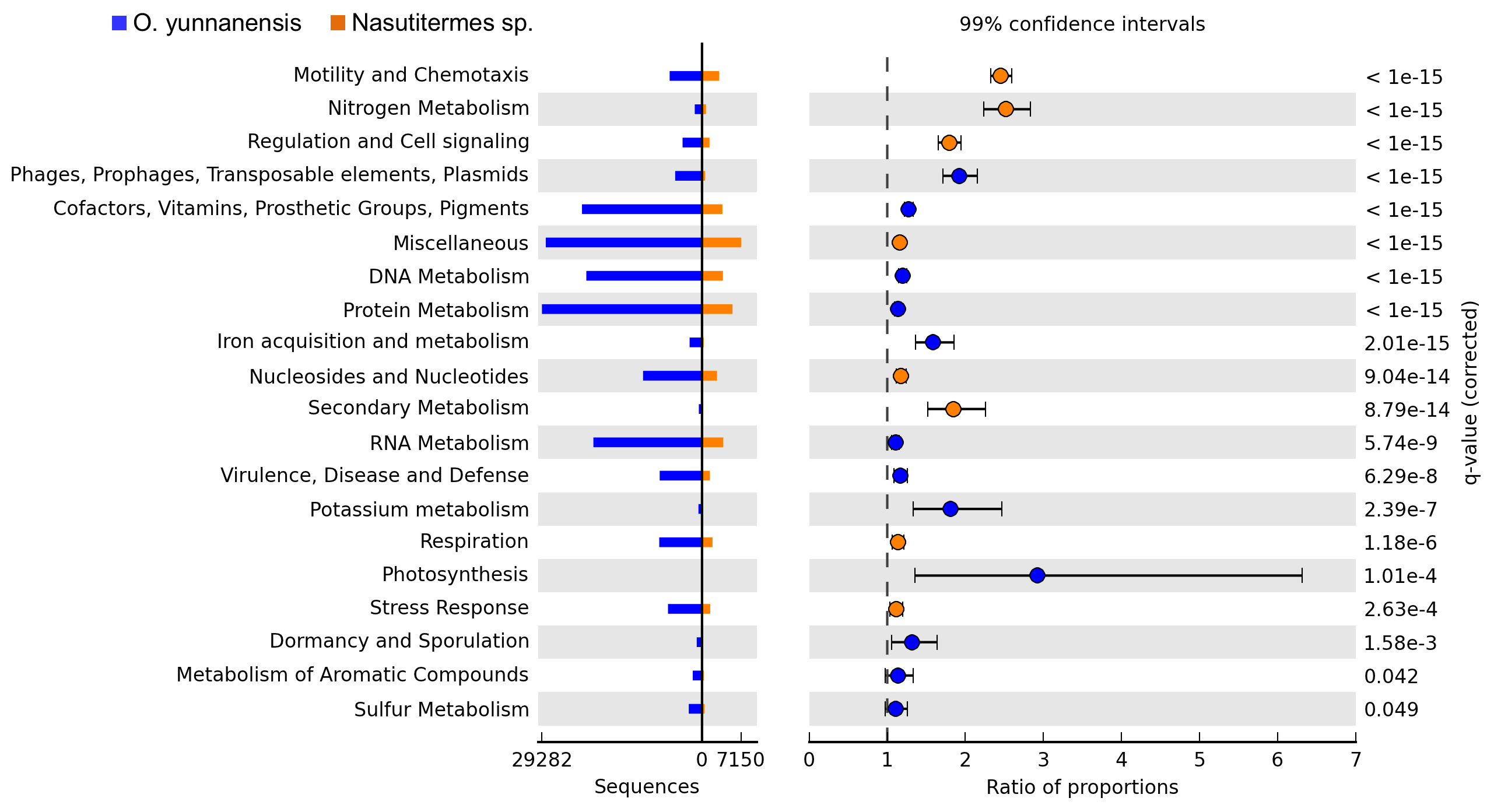


**Figure S4.** **Statistically different SEED classifications between the gut microbiomes of *O. yunnanensis* and the *Nasutitermes* sp. [15].** Classifications statistically overrepresented in the *Odontotermes* metagenome were marked with blue circles, while those statistically overrepresented in the *Nasutitermes* metagenome were marked with orange ones (P<0.05 and Ratio of proportions >1.1 were shown). SEED subsystems-based annotation of both metagenomes was performed as described in the methods. The proportions of environmental gene tags (EGTs) in each classification with respect to the total number of SEED annotated ones in individual metagenome were calculated, based on which ratio of proportions of each classification in the two datasets were further calculated. Gene-centric statistic analysis was performed with two-sided Fisher’s exact test implemented in the STAMP program [33]. P values were corrected by the Benjamini-Hochberg multiple test and confidence intervals were calculated by the Asymptotic method*.*


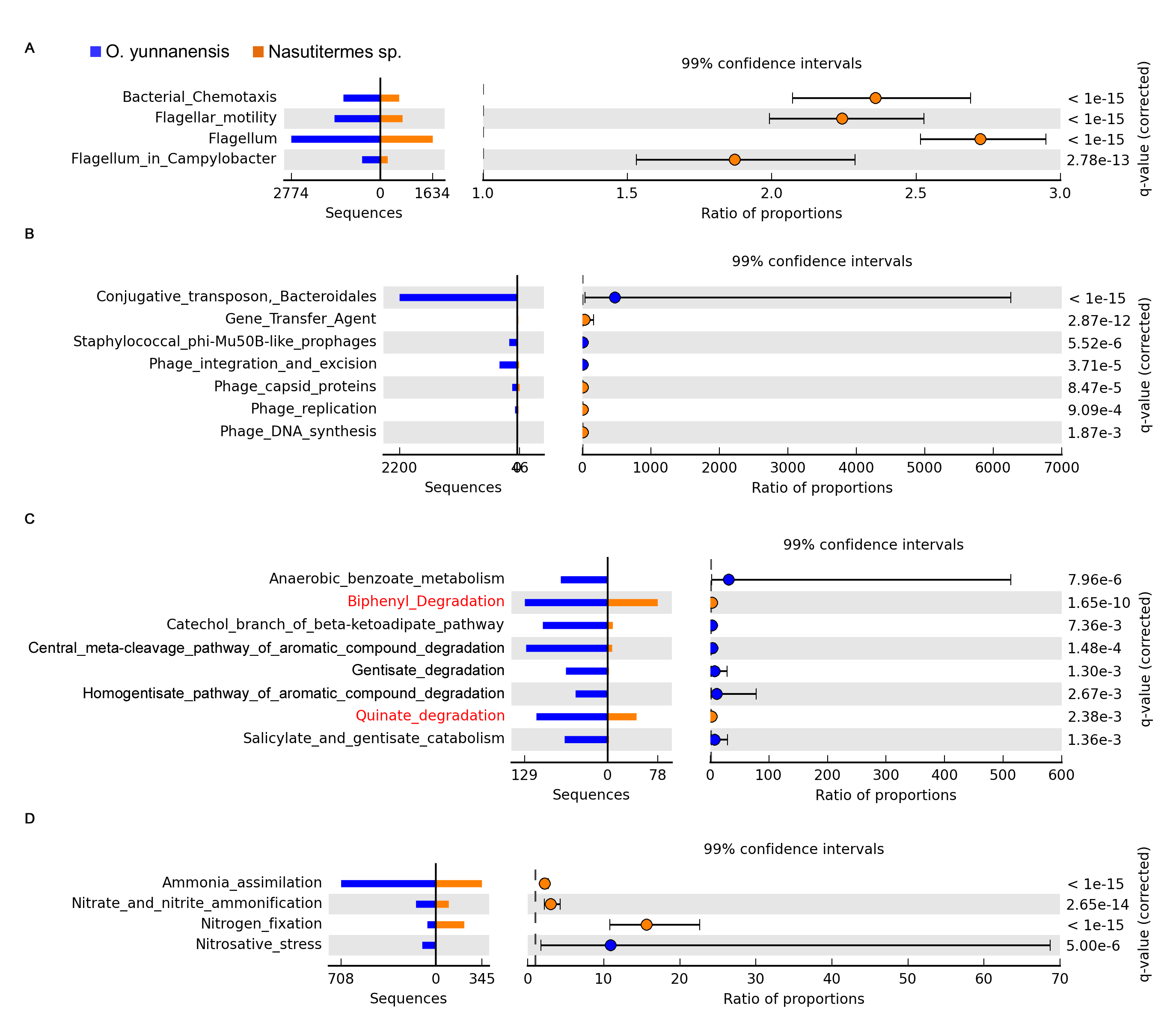


**Figure S5.** **Subsystem distributions in partial statistically different SEED classifications between the gut microbiomes of *O. yunnanensis* and *Nasutitermes* sp.** **[15]** Subsystem distribution of motility and chemotaxis (A), phages, prophages, transposable elements, plasmids (B), metabolism of aromatic compounds (C), and nitrogen metabolism (D). Noticeably, in (C) the two subsystems statistically overrepresented in the *Nasutitermes* metagenome both belong to peripheral pathways of catabolism of aromatic compounds (labeled in red caption), while all subsystems statistically enriched in the *Odontotermes* metagenome belong to metabolism of central aromatic intermediates or aromatic compounds (labeled in black caption, also see Table S5). Statistic analysis was performed with the same procedures and parameters as for Figure S4*.*


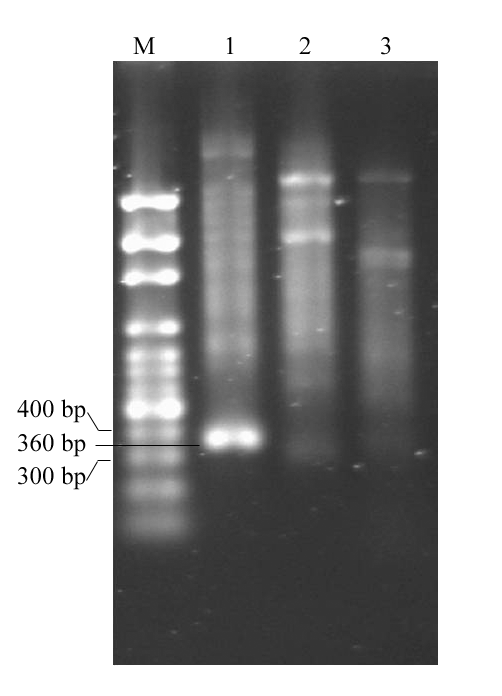


**Figure S6.** **Electrophoresis detection of the PCR amplification product of the *nifH* Gene.** The highly degenerate primers (Pf: 5'-TGXGAXCCYAAZGCYGA-3' X = T or C, Y = A,C,G, or T and Pr: 5'-AWYGCCATCATXTCYCC-3' Z = A or G, W = A,T, or G) designed by Kirshtein et al [57]. were used to amplify the ~360 bp fragment of the *nifH* gene, which encodes the iron protein of nitrogenase that catalyzes N_2_ fixation. M, Quick-Load 100 bp DNA Ladder; 1, amplification products from the whole gut metagenomic DNA of the higher wood-feeding *Globitermes brachycerastes*; 2, amplification products from the whole gut metagenomic DNA of the fungus-cultivating *O. yunnanensis*; 3, amplification products from the whole gut metagenomic DNA of the fungus-cultivating *Macrotermes annandelei.* It revealed that the ~360 bp fragment of the *nifH* gene which encodes the iron protein of nitrogenase could only be amplified from the wood-feeding higher termite species.

Table S1. Phylotype representatives of 16S rRNA sequences obtained from clone library in the whole gut microbiome of *Odontotermes yunnanensis.*

| Category | | Phylotype | No. of clones | Top hit | Accession  no. of top hit | Identity  (%) |
| --- | --- | --- | --- | --- | --- | --- |
| Bacteroidetes | Bacteroidetes_ incertae_sedis | 08112401_Y3GUT_16S_8_78 | 1 | BacIV_clone13 | FN870293 | 92.91 |
|  |  | 08102902-Y3GUT_16S_1_04 | 1 | MgMjD-059 | AB234414 | 98.11 |
|  | Flavobacteria | 08102902-Y3GUT_16S_4_77 | 4 | Chryseobacterium sp. JA37A | EU260141 | 90.91 |
|  | Bacteroidales (*Alistipes*) | 08102902-Y3GUT_16S_2_52 | 3 | MgMjW-65 | AB234444 | 91.84 |
|  |  | 08112401-Y3GUT_16S_6_51 | 9 | MTG-56 | DQ307715 | 95.45 |
|  |  | 08112401-Y3GUT_16S_7_21 | 2 | PeHg49 | FJ374192 | 91.28 |
|  |  | 08112401-Y3GUT_16S_7_44 | 2 | MgMjR-042 | AB234431 | 95.99 |
|  |  | 08102902-Y3GUT_16S_3_54 | 4 | MgMjD-097 | AB234383 | 96.03 |
|  |  | 08102902-Y3GUT_16S_3_75 | 1 | DTB_R15 | EU009783 | 94.6 |
|  |  | 08102902-Y3GUT_16S_1_48 | 1 | PCD-59 | EF608547 | 93.46 |
|  |  | 08102902-Y3GUT_16S_2_26 | 1 | MgMjR-016 | AB234429 | 97.45 |
|  |  | 08102902-Y3GUT_16S_4_19 | 49 | MgMjD-062 | AB234415 | 95.53 |
|  |  | 08102902-Y3GUT_16S_5_17 | 1 | MgMjD-105 | AB234424 | 95.58 |
|  |  | 08102902-Y3GUT_16S_3_78 | 1 | MgMjD-066 | AB234416 | 96.05 |
|  |  | 08112401-Y3GUT_16S_7_83 | 2 | MgMjR-016 | AB234429 | 96.88 |
|  |  | 08102902-Y3GUT_16S_2_68 | 51 | MgMjD-105 | AB234424 | 96.75 |
|  |  | 08102902-Y3GUT_16S_5_76 | 2 | SJTU_C_05_06 | EF404122 | 94.59 |
|  |  | 08102902-Y3GUT_16S_5_40 | 8 | MTG-85 | DQ307722 | 96.41 |
|  |  | 08102902-Y3GUT_16S_4_20 | 7 | BOf8-13 | AB288918 | 97.52 |
|  |  | 08102902-Y3GUT_16S_4_41 | 7 | MgMjD-091 | AB234384 | 96.33 |
|  |  | 08102902-Y3GUT_16S_1_64 | 14 | MgMjD-075 | AB234390 | 96.56 |
|  |  | 08102902-Y3GUT_16S_1_23 | 2 | MgMjD-113 | AB234426 | 95.73 |
|  |  | 08112401_Y3GUT_16S_8_45 | 1 | PeHg49 | FJ374192 | 92.58 |
|  |  | 08102902-Y3GUT_16S_1_90 | 1 | MgMjD-066 | AB234416 | 95.24 |
|  |  | 08102902-Y3GUT_16S_3_51 | 1 | C1-51 | GQ896617 | 93.48 |
|  |  | 08102902-Y3GUT_16S_5_38 | 1 | MTG-12 | DQ307702 | 95.36 |
|  | Bacteroidales (non-*Alistipes)* | 08112401-Y3GUT_16S_6_82 | 4 | BOf3-10 | AB288894 | 88.46 |
|  |  | 08102902-Y3GUT_16S_4_67 | 1 | BOf3-21 | AB288898 | 92.7 |
|  |  | 08102902-Y3GUT_16S_4_37 | 25 | BOf3-21 | AB288898 | 94.74 |
|  |  | 08102902-Y3GUT_16S_5_11 | 14 | C1O | DQ856517 | 95.37 |
|  |  | 08112401-Y3GUT_16S_7_37 | 7 | BOf1-02 | AB288875 | 99.7 |
|  |  | 08102902-Y3GUT_16S_1_88 | 20 | BOf3-21 | AB288898 | 94.82 |
|  |  | 08102902-Y3GUT_16S_2_53 | 1 | BOf2-10 | AB288887 | 91.04 |
|  |  | 08102902-Y3GUT_16S_3_06 | 4 | BOf3-21 | AB288898 | 95.44 |
|  |  | 08112401-Y3GUT_16S_6_23 | 8 | BOf2-10 | AB288887 | 100 |
|  |  | 08112401_Y3GUT_16S_8_43 | 4 | BOf6-19 | AB288907 | 97.19 |
|  |  | 08112401_Y3GUT_16S_8_91 | 8 | Dysgonomonas gadei | AB548675 | 99.5 |
|  |  | 08112401-Y3GUT_16S_6_61 | 3 | BOf7-08 | AB288912 | 98.67 |
|  |  | 08102902-Y3GUT_16S_3_67 | 7 | Rs-096 | AB100459 | 94.33 |
|  |  | 08102902-Y3GUT_16S_2_79 | 1 | 290cost002-P3L-1187 | EF453830 | 91.87 |
|  |  | 08102902-Y3GUT_16S_1_03 | 4 | CNTD10 | HQ728219 | 92.67 |
|  |  | 08112401_Y3GUT_16S_8_31 | 1 | RsStar236 | AB522121 | 96.86 |
|  |  | 08112401-Y3GUT_16S_6_84 | 4 | BOf8-07 | AB288916 | 99.1 |
|  |  | 08112401-Y3GUT_16S_6_45 | 2 | MFC63F06 | FJ823922 | 93.8 |
|  |  | 08102902-Y3GUT_16S_1_08 | 1 | NkW01-004 | AB231048 | 93.46 |
|  |  | 08102902-Y3GUT_16S_4_47 | 1 | RL386_aao88b05 | DQ797187 | 90.47 |
|  |  | 08102902-Y3GUT_16S_4_71 | 2 | Hados.Water.Eubac.8 | AB355049 | 88.7 |
|  |  | 08112401_Y3GUT_16S_8_53 | 1 | 290cost002-P3L-2216 | EF454492 | 93.49 |
|  |  | 08112401-Y3GUT_16S_7_18 | 1 | BOf3-21 | AB288898 | 91.99 |
|  |  | 08102902-Y3GUT_16S_4_30 | 1 | PeH59 | AJ538351 | 93.41 |
| Firmicutes | Erysipelotrichi | 08102902-Y3GUT_16S_1_93 | 2 | BOf1-07 | AB288878 | 97.75 |
|  | Bacilli | 08102902-Y3GUT_16S_5_41 | 8 | BOf1-04 | AB288877 | 97.56 |
|  |  | 08102902-Y3GUT_16S_2_29 | 1 | Lactococcus lactis | FJ915724 | 93.71 |
|  | Clostridiales | 08112401-Y3GUT_16S_6_38 | 1 | MgMjR-012 | AB234479 | 95.58 |
|  |  | 08112401_Y3GUT_16S_8_05 | 2 | Cf4-17 | GQ502579 | 92.03 |
|  |  | 08102902-Y3GUT_16S_2_28 | 1 | MgMjW-18 | AB234504 | 94.44 |
|  |  | 08102902-Y3GUT_16S_5_28 | 7 | MgMjR-068 | AB234449 | 92.49 |
|  |  | 08102902-Y3GUT_16S_2_25 | 1 | BOf10-05 | AB288921 | 92.97 |
|  |  | 08102902-Y3GUT_16S_2_94 | 9 | MgMjW-02 | AB234503 | 97.39 |
|  |  | 08102902-Y3GUT_16S_1_44 | 10 | WF16S_304 | EU939444 | 95.03 |
|  |  | 08112401-Y3GUT_16S_7_73 | 7 | M2PT2-68 | AB192046 | 93.66 |
|  |  | 08112401-Y3GUT_16S_6_47 | 8 | IC21 | FJ798850 | 96.74 |
|  |  | 08112401-Y3GUT_16S_6_86 | 2 | ncd938f01c1 | HM328916 | 91.47 |
|  |  | 08102902-Y3GUT_16S_5_22 | 1 | M77 | AY692049 | 93.03 |
|  |  | 08102902-Y3GUT_16S_3_35 | 2 | Cf4-17 | GQ502579 | 91.23 |
|  |  | 08112401_Y3GUT_16S_8_06 | 1 | MgMjR-031 | AB234485 | 94.37 |
|  |  | 08102902-Y3GUT_16S_1_18 | 1 | ncd230b08c1 | HM262562 | 93.2 |
|  |  | 08112401-Y3GUT_16S_6_04 | 3 | 5 | FJ462126 | 92.66 |
|  |  | 08102902-Y3GUT_16S_3_88 | 1 | dgD-165 | AB264080 | 93.31 |
|  |  | 08112401_Y3GUT_16S_8_37 | 4 | BOf2-18 | AB288889 | 99.4 |
|  |  | 08112401-Y3GUT_16S_6_26 | 2 | BOf6-21 | AB288908 | 96.98 |
|  |  | 08112401_Y3GUT_16S_8_70 | 2 | PeHg80 | FJ374230 | 97.5 |
|  |  | 08102902-Y3GUT_16S_3_01 | 1 | BOf1-09 | AB288879 | 94.87 |
|  |  | 08102902-Y3GUT_16S_5_96 | 1 | M1NP2-89 | AB192023 | 95.53 |
|  |  | 08102902-Y3GUT_16S_1_83 | 2 | 290cost002-P3L-1924 | EF454305 | 94.14 |
|  |  | 08112401-Y3GUT_16S_6_40 | 6 | BOf1-23 | AB288883 | 96.37 |
|  |  | 08102902-Y3GUT_16S_1_89 | 5 | PeHg32 | FJ374219 | 94.66 |
|  |  | 08102902-Y3GUT_16S_2_81 | 1 | PeHg24 | FJ374211 | 96.2 |
|  |  | 08112401_Y3GUT_16S_8_17 | 1 | TSCOR003_H17 | AB486918 | 93.7 |
|  |  | 08112401-Y3GUT_16S_7_29 | 1 | BOf1-09 | AB288879 | 93.98 |
|  |  | 08112401-Y3GUT_16S_7_13 | 2 | ASB34 | FJ947133 | 93.7 |
|  |  | 08112401_Y3GUT_16S_8_88 | 2 | BOf7-19 | AB288914 | 95.25 |
|  |  | 08102902-Y3GUT_16S_2_09 | 1 | dgD-165 | AB264080 | 93.17 |
|  |  | 08102902-Y3GUT_16S_1_16 | 3 | Rs-P74 | AB088981 | 94.89 |
|  |  | 08102902-Y3GUT_16S_1_67 | 1 | BOf4-14 | AB288901 | 92.92 |
|  |  | 08112401-Y3GUT_16S_7_84 | 1 | MgMjR-085 | AB234494 | 95.83 |
|  |  | 08102902-Y3GUT_16S_3_37 | 8 | BOf1-11 | AB288880 | 97.95 |
|  |  | 08112401-Y3GUT_16S_6_72 | 2 | BOf1-01 | AB288874 | 99.46 |
|  |  | 08102902-Y3GUT_16S_3_45 | 1 | A2-26 | GQ897552 | 93.75 |
|  |  | 08112401-Y3GUT_16S_7_95 | 3 | BOf1-09 | AB288879 | 97.37 |
|  |  | 08102902-Y3GUT_16S_3_10 | 1 | HDBW-WB50 | AB237713 | 90.2 |
|  |  | 08102902-Y3GUT_16S_1_75 | 2 | PeHg01 | FJ374210 | 95.74 |
|  |  | 08112401_Y3GUT_16S_8_63 | 2 | BOf5-23 | AB288904 | 97.69 |
|  |  | 08102902-Y3GUT_16S_5_92 | 2 | MgMjW-02 | AB234503 | 97.08 |
|  |  | 08102902-Y3GUT_16S_4_69 | 5 | BS40 | EU358715 | 96.81 |
|  |  | 08102902-Y3GUT_16S_2_47 | 1 | Catabacter sp. | AB490809 | 95.12 |
|  |  | 08112401-Y3GUT_16S_7_60 | 1 | SJTU_C_04_24 | EF404064 | 92.46 |
|  |  | 08102902-Y3GUT_16S_1_74 | 1 | Rs-P74 | AB088981 | 95.84 |
|  |  | 08112401-Y3GUT_16S_6_65 | 1 | HDBW-WB48 | AB237711 | 93.14 |
|  |  | 08102902-Y3GUT_16S_4_32 | 4 | BOf8-01 | AB288915 | 94.76 |
|  |  | 08112401-Y3GUT_16S_7_32 | 2 | MgMjW-18 | AB234504 | 94.07 |
|  |  | 08112401-Y3GUT_16S_7_77 | 1 | PBg1-130 | AY791247 | 95.36 |
|  |  | 08112401_Y3GUT_16S_8_47 | 2 | PeH05 | AJ576325 | 94.99 |
|  |  | 08112401-Y3GUT_16S_7_89 | 1 | HDBW-WB50 | AB237713 | 91.3 |
|  |  | 08112401-Y3GUT_16S_7_48 | 3 | A2-26 | GQ897552 | 93.16 |
|  |  | 08102902-Y3GUT_16S_4_95 | 1 | 1.10135E+12 | EU843957 | 92.48 |
|  |  | 08102902-Y3GUT_16S_1_96 | 1 | M2PT2-68 | AB192046 | 91.24 |
|  |  | 08112401_Y3GUT_16S_8_71 | 2 | BOf2-19 | AB288890 | 96.73 |
|  |  | 08112401-Y3GUT_16S_7_66 | 1 | ncd230b08c1 | HM262562 | 93.86 |
|  |  | 08102902-Y3GUT_16S_2_40 | 2 | MgMjD-072 | AB234466 | 96.59 |
|  |  | 08102902-Y3GUT_16S_5_48 | 1 | BOf2-18 | AB288889 | 97.06 |
|  |  | 08112401_Y3GUT_16S_8_26 | 1 | BOf1-09 | AB288879 | 95.12 |
|  |  | 08102902-Y3GUT_16S_5_39 | 1 | A2-87 | GQ897608 | 94.02 |
|  |  | 08102902-Y3GUT_16S_4_35 | 1 | F24-B10 | AB185589 | 91.83 |
|  |  | 08112401-Y3GUT_16S_6_34 | 1 | PCD-27 | EF608543 | 94.49 |
|  |  | 08112401-Y3GUT_16S_7_82 | 1 | BOf6-04 | AB288905 | 96.77 |
|  |  | 08112401-Y3GUT_16S_6_63 | 1 | BOf6-21 | AB288908 | 95.03 |
|  |  | 08102902-Y3GUT_16S_3_81 | 1 | M1PT4-47 | AB192055 | 93.95 |
|  |  | 08112401_Y3GUT_16S_8_40 | 1 | PCD-66 | EF608549 | 91.67 |
|  |  | 08102902-Y3GUT_16S_2_61 | 1 | aab19h11 | DQ816583 | 90.95 |
|  |  | 08102902-Y3GUT_16S_5_72 | 1 | MB7-1 | DQ453797 | 93.58 |
| Planctomycetes |  | 08112401-Y3GUT_16S_7_43 | 1 | Csp1916 | AM774186 | 93.51 |
|  |  | 08102902-Y3GUT_16S_5_47 | 2 | Csp1104 | AM774187 | 96.86 |
|  |  | 08102902-Y3GUT_16S_3_28 | 3 | MTG-64 | DQ307719 | 97.08 |
|  |  | 08102902-Y3GUT_16S_3_29 | 1 | vadinHA49 | U81766 | 95.11 |
|  |  | 08102902-Y3GUT_16S_4_17 | 1 | MTG-72 | DQ307721 | 97.23 |
|  |  | 08102902-Y3GUT_16S_3_02 | 1 | M1NP1-64 | AB192132 | 91.37 |
|  |  | 08102902-Y3GUT_16S_4_65 | 1 | Csp1104 | AM774187 | 96.65 |
|  |  | 08102902-Y3GUT_16S_3_86 | 2 | Csp1916 | AM774186 | 93.41 |
| Proteobacteria | Alphaproteobacteria | 08102902-Y3GUT_16S_2_65 | 1 | RsStar239 | AB522150 | 92.47 |
|  | Betaproteobacteria | 08102902-Y3GUT_16S_4_86 | 1 | F_SBR_71 | HQ010846 | 93.74 |
|  | Deltaproteobacteria | 08102902-Y3GUT_16S_1_56 | 1 | Cc3-084 | AB299557 | 92.79 |
|  |  | 08102902-Y3GUT_16S_3_56 | 1 | 290cost002-P3L-532 | EF454852 | 92.65 |
|  |  | 08102902-Y3GUT_16S_2_90 | 3 | 290cost002-P3L-1410 | EF453987 | 96.1 |
|  |  | 08102902-Y3GUT_16S_5_29 | 1 | SJTU_C_01_19 | EF403834 | 88.26 |
|  |  | 08102902-Y3GUT_16S_3_91 | 1 | MgMjD-084 | AB234532 | 97.01 |
|  |  | 08102902-Y3GUT_16S_4_92 | 1 | MTG-93 | DQ307726 | 95.07 |
|  |  | 08112401_Y3GUT_16S_8_86 | 1 | PW7 | DQ355176 | 94.85 |
|  |  | 08102902-Y3GUT_16S_1_51 | 1 | BOf2-22 | AB288891 | 98.67 |
|  |  | 08102902-Y3GUT_16S_2_91 | 2 | FC1_16S_61 | EU662469 | 91.85 |
|  |  | 08112401-Y3GUT_16S_7_17 | 1 | MgMjR-025 | AB234535 | 97.72 |
|  |  | 08102902-Y3GUT_16S_5_05 | 1 | Rs-N35 | AB089110 | 93.22 |
|  |  | 08112401-Y3GUT_16S_6_67 | 1 | 290cost002-P3L-532 | EF454852 | 92.22 |
|  |  | 08102902-Y3GUT_16S_5_14 | 1 | 290cost002-P3L-618 | EF454896 | 95.11 |
|  |  | 08102902-Y3GUT_16S_1_37 | 2 | MgMjD-073 | AB234528 | 96.72 |
|  |  | 08112401_Y3GUT_16S_8_24 | 12 | MTG-93 | DQ307726 | 97.1 |
|  |  | 08102902-Y3GUT_16S_2_88 | 7 | BOf1-16 | AB288881 | 97.73 |
|  |  | 08102902-Y3GUT_16S_2_62 | 3 | Rs-N35 | AB089110 | 94.12 |
|  |  | 08102902-Y3GUT_16S_2_04 | 1 | MgMjD-073 | AB234528 | 95.07 |
|  | Epsilonproteobacteria | 08102902-Y3GUT_16S_1_50 | 2 | MgMjR-060 | AB234540 | 98.93 |
|  |  | 08112401_Y3GUT_16S_8_07 | 4 | MgMjD-024 | AB234539 | 98.64 |
| Spirochaetes |  | 08102902-Y3GUT_16S_4_85 | 1 | Spirochaeta sp., za29 | AJ419823 | 94.41 |
|  |  | 08102902-Y3GUT_16S_2_03 | 3 | 290cost002-P3L-1874 | EF454272 | 93.75 |
|  |  | 08102902-Y3GUT_16S_2_48 | 5 | 290cost002-P3L-1874 | EF454272 | 93.28 |
|  |  | 08102902-Y3GUT_16S_3_27 | 8 | MgMjD-093 | AB234370 | 97.97 |
|  |  | 08102902-Y3GUT_16S_4_03 | 3 | MgMjD-028 | AB234361 | 96.73 |
|  |  | 08102902-Y3GUT_16S_1_65 | 2 | MgMjD-028 | AB234361 | 95.63 |
|  |  | 08102902-Y3GUT_16S_2_12 | 7 | M2PT2-79 | AB191944 | 94.22 |
|  |  | 08102902-Y3GUT_16S_1_34 | 2 | Rs-E03 | AB088878 | 93.82 |
|  |  | 08102902-Y3GUT_16S_1_49 | 2 | 290cost002-P3L-1157 | EF453804 | 92.92 |
|  |  | 08102902-Y3GUT_16S_4_90 | 1 | 290cost002-P3L-1645 | EF454106 | 93.93 |
|  |  | 08102902-Y3GUT_16S_2_84 | 1 | Spirochaeta sp., za29 | AJ419823 | 94.56 |
|  |  | 08102902-Y3GUT_16S_3_96 | 1 | MgMjD-028 | AB234361 | 95.41 |
|  |  | 08112401_Y3GUT_16S_8_79 | 1 | 290cost002-P3L-1874 | EF454272 | 92.93 |
|  |  | 08112401_Y3GUT_16S_8_73 | 1 | 290cost002-P3L-1157 | EF453804 | 91.36 |
|  |  | 08102902-Y3GUT_16S_4_58 | 1 | MgMjD-036 | AB234367 | 95.44 |
| Synergistetes |  | 08112401-Y3GUT_16S_2_14 | 2 | 290cost002-P3L-685 | EF454925 | 95.34 |
|  |  | 08112401-Y3GUT_16S_6_77 | 20 | BOf7-03 | AB288910 | 97.38 |
|  |  | 08112401-Y3GUT_16S_7_08 | 1 | 290cost002-P3L-685 | EF454925 | 90.05 |
|  |  | 08112401-Y3GUT_16S_4_51 | 2 | BOf7-03 | AB288910 | 97.2 |
|  |  | 08112401-Y3GUT_16S_1_60 | 1 | MgMjD-049 | AB234552 | 96.84 |
|  |  | 08112401-Y3GUT_16S_2_42 | 1 | Rs-N28 | AB089064 | 90.27 |
|  |  | 08112401-Y3GUT_16S_4_14 | 1 | BOf7-03 | AB288910 | 97.22 |
| Actinobacteria |  | 08102902-Y3GUT_16S_5_55 | 2 | Cf1-12 | GQ502463 | 95.94 |
|  |  | 08102902-Y3GUT_16S_5_13 | 1 | Cf1-06 | GQ502474 | 94.44 |
|  |  | 08102902-Y3GUT_16S_3_12 | 1 | M2PT2-71 | AB192131 | 97.3 |
|  |  | 08112401-Y3GUT_16S_6_29 | 1 | COB P3-21 | AY160874 | 94.15 |
|  |  | 08102902-Y3GUT_16S_5_26 | 1 | Cf1-12 | GQ502463 | 96.68 |
| Candidate division TM7 |  | 08102902-Y3GUT_16S_2_83 | 1 | R-6317 | FJ879268 | 94.22 |
|  |  | 08112401-Y3GUT_16S_7_36 | 1 | Cf8-03 | GQ502661 | 91.08 |
|  |  | 08102902-Y3GUT_16S_4_94 | 1 | 2.20E+13 | EF515301 | 94.52 |
|  |  | 08112401-Y3GUT_16S_7_51 | 2 | Y2 | DQ666092 | 91.44 |
| Chlorobi |  | 08102902-Y3GUT_16S_4_06 | 2 | 290cost002-P3L-2517 | EF454675 | 97.01 |
|  |  | 08102902-Y3GUT_16S_5_12 | 4 | BOf8-10 | AB288917 | 97.15 |
| Elusimicrobia |  | 08102902-Y3GUT_16S_4_29 | 9 | 290cost002-P3L-2571 | EF454709 | 95.81 |
|  |  | 08112401-Y3GUT_16S_6_21 | 1 | 290cost002-P3L-2571 | EF454709 | 96.18 |
| Deferribacteres |  | 08102902-Y3GUT_16S_1_15 | 1 | PBg1-126 | AY791243 | 93.27 |

Table S2. Carbohydrate-active gene modules detected in the gut metagenome of *O.yunnanensis*.

| CAZy family* | Resource^†^ | Known CAZy Activities^§^ | O. yunnanensis |
| --- | --- | --- | --- |
| GH1 | pfam00232 | β-Glucosidase, β-galactosidase, β-mannosidase | 59 |
| GH2 | COG3250^※^ | β-Galactosidase, β-mannosidase, β-glucuronidase | 742 |
| GH3 | pfam00933 | β-Glucosidase, β-xylosidase, α-L-arabinofuranosidase, others | 183 |
| GH4 | pfam02056 | α-Glucosidase, α-galactosidase, α-glucuronidase | 35 |
| GH5 | pfam00150 | Cellulase, β-mannnanse, β-1,3-glucosidase, β-1,4-endoxylanase, others | 67 |
| GH8 | pfam01270 | Cellulase, endo-1,4-β-xylanase, chitosanase, others | 7 |
| GH9 | pfam00759 | Endoglucanase, cellobiohydrolase, β-glucosidase | 59 |
| GH10 | pfam00331 | Xylanase, β-1,3-endoxylanase | 29 |
| GH13 | pfam00128 | α-Amylase, catalytic domain and related enzymes | 202 |
| GH15 | pfam00723 | Glucoamylase, glucodextranase, α-trehalase | 4 |
| GH16 | cd00413 | Licheninase, glucan endo-1,3-beta-D-glucosidase | 34 |
| GH18 | pfam00704 | Chitinase, endo-b-N-acetylglucosaminidase, non-catalytic proteins | 54 |
| GH19 | cd00325 | Chitinase | 6 |
| GH20 | pfam00728 | β-Hexosaminidase, lacto-N-biosidase | 186 |
| GH23 | cd00254 | Lysozyme type G, peptidoglycan lyase | 152 |
| GH24 | cd00737 | Lysozyme | 11 |
| GH25 | pfam01183 | Lysozyme | 17 |
| GH26 | pfam02156 | β-1,3-Xylanase, mannanase | 14 |
| GH27 | pfam02065 | α-Galactosidase | 10 |
| GH28 | pfam00295 | Exo-polygalacturonase, polygalacturonase, rhamnogalacturonase | 81 |
| GH29 | pfam01120 | α-L-fucosidase | 145 |
| GH30 | COG5520 | Glucosylceramidase, β-1,6-glucanase, β-xylosidase, β-glucosidase | 67 |
| GH31 | pfam01055 | α-Glucosidase, α-xylosidase | 122 |
| GH32 | pfam00251 | Invertase, endo-inulinase, endo-levanase, exo-inulinase | 35 |
| GH33 | cd00260 | Sialidase or neuraminidase, trans-sialidase | 10 |
| GH35 | pfam01301 | β-Galactosidase | 20 |
| GH36 | COG3345 | α-Galactosidase | 135 |
| GH37 | pfam01204 | α-Trehalase | 8 |
| GH38 | pfam01074 | α-Mannosidase, mannosyl-oligosaccharide α-1,3-1,6-mannosidase and α-1,3-mannosidase | 30 |
| GH39 | self-built | β-Xylosidase, α-L-iduronidase | 23 |
| GH42 | pfam02449 | β-Galactosidase | 54 |
| GH43 | pfam04616 | Xylanase, β-xylosidase, α-Larabinofuranosidase, arabinanase | 282 |
| GH49 | pfam03718 | Dextranase,isopullulanase, dextran 1,6-α-isomaltotriosidase | 2 |
| GH50 | self-built | β-Agarase | 36 |
| GH51 | COG3534 | Endoglucanase, α-L-arabinofuranosidase | 127 |
| GH53 | pfam07745 | β-1,4-Endogalactanase | 5 |
| GH55 | self-built | Exo-1,3-glucanase, endo-1,3-glucanase | 43 |
| GH57 | pfam03065 | α-Amylase, 4-α-glucanotransferase, α-galactosidase | 107 |
| GH59 | pfam02057 | Galactocerebrosidase | 10 |
| GH63 | PRK10137 | Processing α-glucosidase,α-1,3-glucosidase,α-glucosidase | 21 |
| GH65 | pfam03632 | Trehalase, maltosephosphorylase, trehalosephosphorylase | 18 |
| GH66 | self-built | Cycloisomaltooligosaccharide glucanotransferase; dextranase | 5 |
| GH67 | COG3661 | α-Glucuronidase | 36 |
| GH70 | pfam02324 | Dextransucrase, alternansucrase, reuteransucrase | 1 |
| GH73 | pfam01832 | Peptidoglycan hydrolase with endo-β-N-acetylglucosaminidase specificity | 40 |
| GH74 | smart00602 | Endoglucanase; oligoxyloglucan reducing end-specific cellobiohydrolase; xyloglucanase | 23 |
| GH76 | pfam03663 | α-1,6-Mannanase | 22 |
| GH77 | pfam02446 | 4-α-Glucanotransferase, amylomaltase | 126 |
| GH78 | pfam05592 | α-L-rhamnosidase | 225 |
| GH81 | pfam03639 | Endo-β-1,3-glucanase | 7 |
| GH82 | COG5434 | Ι-carrageenase | 7 |
| GH85 | pfam03644 | Endo-β-N-acetylglucosaminidase | 2 |
| GH87 | self-built | Mycodextranase, α-1,3-glucanase | 6 |
| GH88 | pfam07470 | D-4,5-Unsaturated b-glucuronyl hydrolase | 56 |
| GH89 | pfam05089 | α-N-acetylglucosaminidase | 17 |
| GH91 | self-built | Inulin lyase; difructofuranose 1,2':2,3' dianhydride hydrolase | 2 |
| GH92 | pfam07971 | Mannosyl-oligosaccharide α-1,2-mannosidase, α-1,3-mannosidase, and α-1,6-mannosidase; α-1,2-mannosidase; α-1,3-mannosidase, others | 427 |
| GH93 | self-built | Exo-1,5-α-L-arabinanase | 9 |
| GH94 | COG3459^＃^ | Cellobiose phosphorylase, chitobiose phosphorylase | 60 |
| GH95 | self-built | α-1,2-L-fucosidase, α-L-fucosidase | 195 |
| GH97 | pfam10566 | α-Glucosidase, α-galactosidase | 162 |
| GH99 | self-built | Glycoprotein endo-α-1,2-mannosidase | 19 |
| GH101 | self-built | Endo-α-N-acetylgalactosaminidase | 1 |
| GH102 | pfam03562 | Peptidoglycan lytic transglycosylase | 3 |
| GH103 | TIGR02283 | Peptidoglycan lytic transglycosylase | 20 |
| GH105 | pfam07470 | Unsaturated rhamnogalacturonyl hydrolase | 76 |
| GH106 | self-built | α-L-Rhamnosidase | 161 |
| GH107 | self-built | Sulfated fucan endo-1,4-fucanase | 1 |
| GH108 | pfam05838 | N-acetylmuramidase | 6 |
| GH109 | pfam01408 | α-N-Acetylgalactosaminidase | 249 |
| GH110 | self-built | α-Galactosidase, α-1,3-galactosidase | 12 |
| GH112 | pfam09508 | Lacto-N-biose phosphorylase or galacto-N-biose phosphorylase, D-galactosyl-1,4-L-rhamnose phosphorylase | 16 |
| GH113 | self-built | β-mannanase | 2 |
| GH114 | pfam03537 | Endo-α-1,4-polygalactosaminidase | 5 |
| GH115 | self-built | Xylan α-1,2-glucuronidase, α-(4-O-methyl)-glucuronidase | 31 |
| GH116 | pfam04685 | Acid β-glucosidase, β-glucosidase, β-xylosidase | 3 |
| GH117 | pfam04616 | α-1,3-L-neoagarooligosaccharide hydrolase ; α-1,3-L-neoagarobiase | 13 |
| GH119 | self-built | α-Amylase | 12 |
| GH120 | pfam07602 | β-Xylosidase | 2 |
| GH121 | self-built | β-L-arabinobiosidase | 5 |
| GH123 | self-built | Glycosphingolipid β-N-acetylgalactosaminidase | 20 |
| GH124 | GH124 | Endoglucanase | 4 |
| GH125 | pfam06824 | Exo-α-1,6-mannosidase | 56 |
| GH126 | nil | α-Amylase | 3 |
| GH127 | nil | β-L-arabinofuranosidase | 143 |
| GH128 | nil | β-1,3-Glucanase | 1 |
| GH129 | nil | α-N-acetylgalactosaminidase | 5 |
| GH130 | nil | 1-β-D-mannopyranosyl-4-D-glucopyranose:phosphate α-D-mannosyltransferase | 35 |
| CBM2 | pfam00553 | Cellulose-, xylan-, chitin- and mannan-binding domain | 8 |
| CBM4 | pfam02018 | Cellulose-, β-1,3-glucan- and Xylan-binding domain | 14 |
| CBM5 | pfam02839 | Chitin-binding domain | 18 |
| CBM6 | pfam03422 | Cellulose-, xylan-binding domain | 26 |
| CBM8 | self-built | Cellulose-binding domain | 1 |
| CBM9 | pfam06452 | Cellulose-, xylan-binding domain | 22 |
| CBM11 | pfam03425 | β-1,4-Glucan-, and β-1,3-1,4-mixed linked glucans-binding domain | 1 |
| CBM12 | pfam02839 | Chitin-binding domain | 11 |
| CBM13 | pfam00652 | Ricin-type beta-trefoil lectin domain | 15 |
| CBM14 | pfam01607 | Chitin-binding Peritrophin-A domain | 4 |
| CBM16 | pfam02018 | Cellulose- and glucomannan-binding domain | 6 |
| CBM20 | pfam00686 | Starch-binding domain | 11 |
| CBM22 | pfam02018 | Xylan-, β-1,3-1,4-glucans-binding domain | 3 |
| CBM26 | self-built | Starch-binding domain | 2 |
| CBM30 | pfam02927 | Cellulose-binding domain | 4 |
| CBM32 | pfam00754 | Galactose-, lactose-, polygalacturonic acid- and LacNAc-binding domain | 59 |
| CBM33 | pfam03067 | Chitin-binding domain | 1 |
| CBM34 | cd02857 | α-Amylase, N-terminal ig-like domain | 8 |
| CBM35 | pfam03422 | Xylan-, mannans-, mannooligosaccharides- and β-galactan-binding | 9 |
| CBM37 | smart00060 | Xylan-, chitin-, cellulose-binding domain | 18 |
| CBM40 | pfam02973 | Sialidase, N-terminal domain | 7 |
| CBM42 | pfam05270 | Arabinofuranose-binding domain | 1 |
| CBM44 | pfam00801 | Cellulose- and xyloglucan-binding domain | 5 |
| CBM46 | pfam03442 | Cellulose-binding domain | 5 |
| CBM48 | pfam02922 | Glycogen-binding domain | 64 |
| CBM50 | pfam01476 | Chitopentaose-binding domain | 173 |
| CBM51 | pfam08305 | Galactose- and blood group A/B-antigens-binding domain | 6 |
| CBM54 | self-built | Xylan-binding, yeast cell wall glucan- and chitin-binding domain | 6 |
| CBM56 | self-built | β-1,3-Glucan-binding domain | 28 |
| CBM57 | pfam11721 | Attached to various glycosidases | 1 |
| CBM59 | self-built | Mannan-, xylan-, and cellulose-binding domain | 2 |
| CBM61 | self-built | β-1,4-Galactan-binding domain, dextran-binding domain | 3 |
| CBM62 | self-built | Binds galactose moieties found on xyloglucan, arabinogalactan and galactomannan | 19 |
| CE1 | pfam00756 | Acetyl xylan esterase, feruloyl esterase, carboxylesterase | 223 |
| CE2 | cd01831 | Acetyl xylan esterase | 6 |
| CE3 | cd01833 | Acetyl xylan esterase | 26 |
| CE4 | pfam01522 | Chitin, chitooligosaccharide, peptidoglycan GlcNAc and N-acetylmuramic acid deacetylase | 81 |
| CE5 | pfam01083 | Acetyl xylan esterase; cutinase | 1 |
| CE6 | pfam03629 | Acetyl xylan esterase | 17 |
| CE7 | pfam05448 | Acetyl xylan esterase, cephalosporin-C deacetylase | 58 |
| CE8 | pfam01095 | Pectin methylesterase | 30 |
| CE9 | cd00854 | N-acetylglucosamine 6-phosphate deacetylase | 285 |
| CE11 | pfam03331 | UDP-3-0-acyl N-acetylglucosamine deacetylase | 109 |
| CE12 | cd01821 | Pectin acetylesterase, rhamnogalacturonan acetylesterase, acetyl xylan esterase | 27 |
| CE14 | pfam02585 | N-acetyl-1-D-myo-inosityl-2-amino-2-deoxy-α-D-glucopyranoside deacetylase, diacetylchitobiose deacetylase | 16 |
| CE15 | self-built | 4-O-methyl-glucuronoyl methylesterase | 31 |
| PL1 | smart00656 | Pectate lyase, exo-pectate lyase, pectin lyase | 15 |
| PL4 | self-built | Rhamnogalacturonan lyase | 16 |
| PL6 | self-built | Alginate lyase; chondroitinase B | 2 |
| PL8 | pfam02278 | Hyaluronate lyase, chondroitin AC lyase, xanthan lyase | 5 |
| PL9 | self-built | Pectate lyase, exopolygalacturonate lyase, thiopeptidoglycan lyase | 14 |
| PL10 | pfam09492 | Pectate lyase | 14 |
| PL11 | self-built | Rhamnogalacturonan lyase, exo-unsaturated rhamnogalacturonan lyase | 36 |
| PL12 | pfam07940 | Harin-sulfate lyase | 11 |
| PL13 | self-built | Heparin lyase | 1 |
| PL15 | pfam07940 | Oligo-alginate lyase | 2 |
| PL17 | pfam07940 | Aginate lyase | 3 |
| PL21 | pfam07940 | Heparin lyase, heparin-sulfate lyase, acharan-sulfate lyase | 2 |
| PL22 | TIGR02800 | Oligogalacturonate lyase / oligogalacturonide lyase | 60 |
| Total |  |  | 7236 |

^*^ The carbohydrate-active enzyme database (CAZy), [http://www.CAZy.org](http://www.CAZy.org/).

^†^ Carbohydrate-active enzymes were detected with the set of CAZy family-specific HMMs defined by Yin et al. [24], as deposited on dbCAN (<http://csbl.bmb.uga.edu/dbCAN/>), for the BLAST searches (E ≤ 10^-4^).

^§^ Known CAZy activities were given according to the CAZy database.

^※^ According to dbCAN, the HMM for GH2 was COG3250, which corresponds to Pfam families including Glyco_hydro_2 (pfam00703), Glyco_hydro_2_C (pfam02836) and Glyco_hydro_2_N (pfam02837).

^＃^ According to dbCAN, the HMM for GH94 was COG3459, which corresponds to Pfam families including PF06204 (CBM_X), PF06165 (Glyco_transf_36) and PF06205 (GT36_AF).

One GH48 read (E-value: 3.8e-05) was identified with the set of dbCAN HMMs but proved to be a false positive by manual curation and was excluded from Table S2.

Table S3. Domains often associated with GH catalytic domains detected in the gut metagenome of *O.yunnanensis*.

| Pfam HMM Name | Pfam Accession | Pfam Description | Associated CAZy family^*^ | *O. yunnanensis* |
| --- | --- | --- | --- | --- |
| 3D | PF06725 | 3D domain | GH102 | 14 |
| Alpha-amylase_C | PF02806 | Alpha amylase, C-terminal all-beta domain | GH13 | 38 |
| Alpha-amylase_N | PF02903 | Alpha amylase, N-terminal ig-like domain | GH13 | 2 |
| Alpha-L-AF_C | PF06964 | Alpha-L-arabinofuranosidase C-terminus | GH51 | 45 |
| Alpha-mann_mid | PF09261 | Alpha mannosidase, middle domain | GH38 | 11 |
| Bac_rhamnosid_N | PF08531 | Alpha-L-rhamnosidase N-terminal domain | GH78 | 77 |
| Bgal_small_N | PF02929 | Beta galactosidase small chain | GH2 | 103 |
| Big_1 | PF02369 | Bacterial Ig-like domain (group 1) |  | 86 |
| Big_2 | PF02368 | Bacterial Ig-like domain (group 2) |  | 716 |
| Big_3 | PF07523 | Bacterial Ig-like domain (group 3) |  | 44 |
| CBM_X | PF06204 | Putative carbohydrate binding domain | GH94 | 5 |
| CelD_N | PF02927 | N-terminal ig-like domain of cellulase | GH9 | 18 |
| CHB_HEX | PF03173 | Putative carbohydrate binding domain | GH20 | 1 |
| CHB_HEX_C | PF03174 | Chitobiase/beta-hexosaminidase C-terminal domain | GH20 | 27 |
| ChiC | PF06483 | Chitinase C |  | 1 |
| DUF1957 | PF09210 | Domain of unknown function (DUF1957) | GH57 | 15 |
| fn3 | PF00041 | Fibronectin type III domain |  | 106 |
| GDE_C | PF06202 | Amylo-alpha-1,6-glucosidase |  | 44 |
| Glyco_hydro_20b | PF02838 | Glycosyl hydrolase family 20, domain 2 | GH20 | 24 |
| Glyco_hydro_3_C | PF01915 | Glycosyl hydrolase family 3 C terminal domain | GH3 | 147 |
| Glyco_hydro_32C | PF08244 | Glycosyl hydrolases family 32 C terminal | GH32 | 5 |
| Glyco_hydro_38C | PF07748 | Glycosyl hydrolases family 38 C-terminal domain | GH38 | 38 |
| Glyco_hydro_42C | PF08533 | Beta-galactosidase C-terminal domain | GH42 | 6 |
| Glyco_hydro_42M | PF08532 | Beta-galactosidase trimerisation domain | GH42 | 42 |
| Glyco_hydro_65C | PF03633 | Glycosyl hydrolase family 65, C-terminal domain | GH65 | 2 |
| Glyco_hydro_65N | PF03636 | Glycosyl hydrolase family 65, N-terminal domain | GH65 | 30 |
| He_PIG | PF05345 | Putative Ig domain |  | 157 |
| MLTD_N | PF06474 | MLTD_N | GH23 | 1 |
| PG_binding_1 | PF01471 | Putative peptidoglycan binding domain | GH103 | 51 |
| PG_binding_3 | PF09374 | Predicted Peptidoglycan domain | GH108 | 1 |
| Rod-binding | PF10135 | Rod binding protein | GH73 | 10 |
| SBP_bac_3 | PF00497 | Bacterial extracellular solute-binding proteins, family 3 | GH23 | 155 |
| Sialidase | PF02973 | Sialidase, N-terminal domain | GH33 | 8 |
| TIG | PF01833 | IPT/TIG domain |  | 62 |
| ChitinaseA_N | PF08329 | Chitinase A, N-terminal domain | GH18 | 7 |
| Cohesin | PF00963 | Cohesin domain |  | 7 |
| Dockerin_1 | PF00404 | Dockerin type I repeat |  | 1 |
| SLH | PF00395 | S-layer homology domain |  | 74 |

^*^ Domains often associated with GH catalytic domains were summarized according to Park et al. [25] and Warnecke et al [15]. Since Pfam families PF00703 (Glyco_hydro_2) and PF02837 (Glyco_hydro_2_N) were included in COG3250 for GH2, and PF06204 (CBM_X), PF06165 (Glyco_transf_36) and PF06205 (GT36_AF) were included in COG3459 for GH94, as seen the footnote of table S2, they were not listed here.

Table S4. Comparison of CAZy profiles of the whole gut metagenome of *O. yunnanensis* with those of the leaf cutter ant fungus garden [18], wallaby foregut [17], and the wood-feeding *Nasutitermes sp.* hindgut [15].

| **CAZy**  **family** | ***O. yunnanensis***  **(fungus-cultivating termite whole gut)** | ***At. cephalotes***  **(Leaf cutter ant fungus garden)** | **Wallaby foregut** | ***Nasutitermes* sp.**  **(wood-feeding termite hindgut)** |
| --- | --- | --- | --- | --- |
| **Cellulases and endohemicellulases** |  |  |  |  |
| GH5 | 67 | 2 | 20 | 97 |
| GH6 | 0 | 2 | 0 | 0 |
| GH8 | 7 | 13 | 2 | 17 |
| GH9 | 59 | 0 | 4 | 39 |
| GH10 | 29 | 3 | 18 | 92 |
| GH11 | 0 | 0 | 0 | 18 |
| GH26 | 14 | 0 | 8 | 19 |
| GH44 | 0 | 0 | 0 | 5 |
| GH45 | 0 | 0 | 0 | 6 |
| GH74 | 23 | 0 | 0 | 1 |
| GH113 | 2 | 0 | 1 | 4 |
| GH124 | 4 | nr | nr | nr |
| Total | 205 (2.8%) | 20(3.1%) | 53(5.2%) | 298(20.5%) |
| **Pectinases** |  |  |  |  |
| GH28 | 81 | 3 | 10 | 13 |
| GH105 | 76 | 14 | 5 | 9 |
| CE8 | 30 | 7 | 4 | 0 |
| CE12 | 27 | 0 | 6 | 3 |
| PL1 | 15 | 1 | 0 | 1 |
| PL2 | 0 | 3 | 1 | 1 |
| PL3 | 0 | 0 | 1 | 0 |
| PL4 | 16 | 1 | 1 | 0 |
| PL9 | 14 | 8 | 8 | 4 |
| PL10 | 14 | 1 | 0 | 0 |
| PL11 | 36 | 0 | 0 | 0 |
| PL22 | 60 | nr | nr | nr |
| Total | 369(5.1%) | 38(6.0%) | 36(3.5%) | 31(2.1%) |
| **Debranching enzymes and Oligosaccharide-degrading enzymes** |  |  |  |  |
| GH1 | 59 | 77 | 84 | 27 |
| GH2 | 742 | 8 | 33 | 30 |
| GH3 | 183 | 29 | 98 | 108 |
| GH4 | 35 | 35 | 3 | 17 |
| GH27 | 10 | 0 | 8 | 4 |
| GH29 | 145 | 0 | 5 | 12 |
| GH30 | 67 | 0 | 6 | 9 |
| GH31 | 122 | 37 | 30 | 36 |
| GH35 | 20 | 0 | 10 | 6 |
| GH36 | 135 | 9 | 29 | 4 |
| GH39 | 23 | 0 | 3 | 11 |
| GH42 | 54 | 3 | 17 | 34 |
| GH43 | 282 | 11 | 47 | 57 |
| GH51 | 127 | 0 | 18 | 26 |
| GH52 | 0 | 0 | 0 | 3 |
| GH53 | 5 | 0 | 3 | 5 |
| GH67 | 36 | 0 | 0 | 6 |
| GH78 | 225 | 4 | 52 | 7 |
| GH93 | 9 | 2 | 0 | 0 |
| GH94 | 60 | 0 | 37 | 135 |
| GH97 | 162 | 0 | 15 | 0 |
| GH106 | 161 | 0 | 4 | 2 |
| GH115 | 31 | 0 | 5 | 17 |
| GH116 | 3 | nr | nr | nr |
| GH120 | 2 | nr | nr | nr |
| GH121 | 5 | nr | nr | nr |
| GH127 | 143 | nr | nr | nr |
| CE1 | 223 | 0 | 0 | 0 |
| CE2 | 6 | 0 | 0 | 4 |
| CE3 | 26 | 0 | 0 | 0 |
| CE5 | 1 | 0 | 0 | 0 |
| CE6 | 17 | 0 | 2 | 2 |
| CE7 | 58 | 0 | 3 | 0 |
| Total | 3177(43.9%) | 215(33.8%) | 512(49.8%) | 562(38.7%) |
| **CAZy families processing fungal cell wall** |  |  |  |  |
| GH18 | 54 | 15 | 6 | 27 |
| GH19 | 6 | 6 | 0 | 1 |
| GH20 | 186 | 5 | 13 | 18 |
| GH38 | 30 | 4 | 3 | 26 |
| GH55 | 43 | 0 | 0 | 3 |
| GH76 | 22 | 0 | 0 | 0 |
| GH81 | 7 | 0 | 0 | 0 |
| GH85 | 2 | 0 | 0 | 0 |
| GH92 | 427 | 0 | 17 | 2 |
| GH99 | 19 | 0 | 0 | 0 |
| GH125 | 56 | nr | nr | nr |
| GH128 | 1 | nr | nr | nr |
| CE4 | 81 | 16 | 31 | 33 |
| Total | 934(12.9%) | 46(7.2%) | 70(6.8%) | 110(7.6%) |

The CAZymes data of metagenomes of leaf cutter ant fungus garden (*At. cephalotes* combined) [18], wallaby foregut [17], and the wood-feeding *Nasutitermes sp.* hindgut [15] were presented using the format and data described in [18] and its supplemental materials, with the CAZy families grouped according to their major functional roles. The numbers in parentheses represent the percentages of these groups against the total number of CAZymes identified in individual metagenomes, nr: not reported.

Table S5. Distribution of statistically different subsystems in metabolism of aromatic compounds between the gut microbiomes of *O. yunnanensis* and the *Nasutitermes sp.* [15].

| Subclassification | Subsystem | *O.yunnanensis*^*^ | *Nasutitermes sp*^§^ | Effect size^＃^ |
| --- | --- | --- | --- | --- |
| Peripheral pathways for catabolism of aromatic compounds | Biphenyl Degradation^†^ | 129 | 78 | 0.36 |
|  | Quinate degradation^†^ | 111 | 45 | 0.53 |
| Anaerobic degradation of aromatic compounds | Anaerobic benzoate metabolism^¶^ | 73 | 0 | 31.8 |
| Metabolism of Aromatic Compounds | Gentisate degradation^¶^ | 65 | 2 | 7.03 |
| Metabolism of central aromatic intermediates | Salicylate and gentisate catabolism^¶^ | 67 | 2 | 7.25 |
|  | Catechol branch of beta-ketoadipate pathway^¶^ | 101 | 8 | 2.73 |
|  | Central meta-cleavage pathway of aromatic compound degradation^¶^ | 127 | 7 | 3.92 |
|  | Homogentisate pathway of aromatic compound degradation^¶^ | 50 | 1 | 10.8 |

^*^ EGTs in the gut metagenome of *O.yunnanensis.*

^§^ EGTs in the gut metagenome of *Nasutitermes sp.*

^＃^Effect size was calculated by the STAMP program [33] and was defined as the ratio of proportions of each subsystem in *O. yunnanensis* divide that in the *Nasutitermes* sp*.* Statistical analysis was performed with the two-sided Fisher’s exact test (P<0.01, 99% confidence intervals), P values were corrected by the Benjamini-Hochberg multiple test and confidence intervals were calculated by the Asymptotic method*.*

^†^ Subsystems overrepresented in the wood-feeding *Nasutitermes* gut microbiome, as calculated and presented in Fig. S5C.

^¶^ Subsystems overrepresented in the fungus-cultivating *Odontotermes* gut microbiome, as calculated and presented in Fig. S5C.

Table S6. Composition of statistically different subsystems in nitrogen metabolism between the gut microbiomes of *O. yunnanensis* and the *Nasutitermes* sp. [15].

| Subsystem | Function role | *O.yunnanensis*^*^ | *Nasutitermes sp.* ^§^ |
| --- | --- | --- | --- |
| Ammonia assimilation**^†^** |  | 708 | 345 |
|  | [Protein-PII] uridylyltransferase (EC 2.7.7.59) | 2 |  |
|  | Ammonium transporter | 3 | 29 |
|  | Ammonium transporter family | 1 |  |
|  | ammonium/methylammonium permease | 2 | 3 |
|  | Glutamate synthase [NADPH] large chain (EC 1.4.1.13) | 142 | 77 |
|  | Glutamate synthase [NADPH] small chain (EC 1.4.1.13) | 158 | 47 |
|  | Glutamate synthase [NADPH] putative GlxC chain (EC 1.4.1.13) | 1 |  |
|  | Glutamine synthetase type I (EC 6.3.1.2) | 95 | 3 |
|  | Glutamine synthetase type III, GlnN (EC 6.3.1.2) | 247 | 34 |
|  | Glutamine synthetase, clostridia type (EC 6.3.1.2) | 9 |  |
|  | Nitrogen regulation protein NR(I) | 27 | 5 |
|  | Nitrogen regulatory protein P-II | 19 | 59 |
|  | Glutamate synthase, alpha subunit domain protein |  | 46 |
|  | Glutamine amidotransferase, class-II |  | 42 |
|  | Glutamate-ammonia-ligase adenylyltransferase (EC 2.7.7.42) | 2 |  |
| Nitrate and nitrite ammonification**^†^** |  | 148 | 97 |
|  | Assimilatory nitrate reductase large subunit (EC:1.7.99.4) | 4 |  |
|  | Cytochrome c nitrite reductase, small subunit NrfH | 15 |  |
|  | Cytochrome c552 precursor (EC 1.7.2.2) | 14 | 1 |
|  | Ferredoxin-type protein NapF (periplasmic nitrate reductase) | 37 |  |
|  | Ferredoxin-type protein NapG (periplasmic nitrate reductase) |  | 1 |
|  | Nitrate ABC transporter, ATP-binding protein | 3 |  |
|  | Nitrate ABC transporter, nitrate-binding protein | 1 |  |
|  | Nitrate/nitrite response regulator protein | 12 | 3 |
|  | Nitrate/nitrite sensor protein (EC 2.7.3.-) | 4 |  |
|  | Nitrate/nitrite transporter | 2 | 1 |
|  | Nitrite reductase [NAD(P)H] large subunit (EC 1.7.1.4) | 4 |  |
|  | Nitrite reductase probable [NAD(P)H] subunit (EC 1.7.1.4) | 17 | 62 |
|  | Nitrite reductase probable electron transfer 4Fe-S subunit (EC 1.7.1.4) | 13 | 21 |
|  | Nitrite transporter NirC | 1 | 3 |
|  | NrfC protein | 3 |  |
|  | Periplasmic nitrate reductase precursor (EC 1.7.99.4) | 8 | 2 |
|  | Polyferredoxin NapH (periplasmic nitrate reductase) | 8 | 3 |
|  | Respiratory nitrate reductase alpha chain (EC 1.7.99.4) | 2 |  |
| Nitrogen fixation**^†^** |  | 63 | 212 |
|  | Cysteine desulfurase (EC 2.8.1.7), NifS subfamily | 19 | 7 |
|  | Iron-sulfur cluster assembly scaffold protein NifU | 13 | 4 |
|  | Nitrogenase (iron-iron) alpha chain (EC 1.18.6.1) | 2 |  |
|  | Nitrogenase (iron-iron) beta chain (EC 1.18.6.1) | 1 |  |
|  | Nitrogenase (iron-iron) transcriptional regulator | 1 |  |
|  | Nitrogenase (molybdenum-iron) alpha chain (EC 1.18.6.1) | 1 | 29 |
|  | Nitrogenase (molybdenum-iron) beta chain (EC 1.18.6.1) | 1 | 23 |
|  | Nitrogenase (molybdenum-iron) reductase and maturation protein NifH | 3 | 11 |
|  | Nitrogenase (molybdenum-iron)-specific transcriptional regulator NifA | 8 | 2 |
|  | Nitrogenase FeMo-cofactor scaffold and assembly protein NifE | 3 | 18 |
|  | Nitrogenase FeMo-cofactor synthesis FeS core scaffold and assembly protein NifB | 6 | 37 |
|  | Nitrogenase vanadium-cofactor synthesis protein VnfN | 4 | 17 |
|  | Nitrogenase vanadium-cofactor synthesis protein VnfE |  | 11 |
|  | Nitrogenase (vanadium-iron) reductase and maturation protein VnfH | 1 |  |
|  | Nitrogenase FeMo-cofactor scaffold and assembly protein NifN |  | 31 |
|  | NifB-domain protein, type 2 |  | 5 |
|  | Homocitrate synthase alpha subunit (EC 2.3.3.14) |  | 8 |
|  | Homocitrate synthase omega subunit (EC 2.3.3.14) |  | 6 |
|  | AnfO protein, required for Mo- and V-independent nitrogenase |  | 3 |
| Nitrosative stress^＃^ |  | 101 | 2 |
|  | Ferredoxin 3 fused to uncharacterized domain | 37 |  |
|  | Functional role page for Anaerobic nitric oxide reductase transcription regulator NorR | 4 |  |
|  | Hcp transcriptional regulator HcpR (Crp/Fnr family) | 15 |  |
|  | Hydroxylamine reductase (EC 1.7.-.-) | 42 |  |
|  | Nitric-oxide reductase (EC 1.7.99.7), quinol-dependent | 2 | 2 |
|  | Nitrite-sensitive transcriptional repressor NsrR | 1 |  |

^*^ EGTs in the gut metagenome of *O.yunnanensis.*

^§^ EGTs in the gut metagenome of *Nasutitermes sp.*

^†^ Subsystems overrepresented in the wood-feeding *Nasutitermes* gut microbiome, as calculated and presented in Fig. S5D.

^＃^ Subsystem overrepresented in the present fungus-cultivating *Odontotermes* gut microbiome, as calculated and presented in Fig. S5D.
